# Comparative Analysis of Fungal Spore Flora Among Birds, Insects and Air in a Temperate Japanese Forest

**DOI:** 10.1002/ece3.72929

**Published:** 2026-01-11

**Authors:** Rohit Bangay, Shunsuke Matsuoka, Nobuko Tuno

**Affiliations:** ^1^ Division of Natural System, Graduate School of Natural Science & Technology Kanazawa University Kanazawa Ishikawa Japan; ^2^ Field Science Education and Research Centre Kyoto University Kyoto Japan

**Keywords:** bioaerosols, forest ecology, fungal–bird interactions, mycobiome, mycophagy, pathogens

## Abstract

Fungi play critical roles in ecosystem functioning, yet the mechanisms underlying their spore dispersal, especially via animal vectors, remain underexplored. We investigated the potential of birds and insects as fungal spore vectors in Japanese temperate forests by analysing fungal communities on the feathers of 14 bird species and within 158 insects collected from fungal fruiting bodies. Comparisons were also made with air samples collected from the surrounding forest (*n =* 124). Microscopy and next generation sequencing (NGS) revealed fungal spores on all bird feather samples, with *Cladosporium* and *Penicillium* most abundant when analysed microscopically, and identified 39 assigned fungal species across 381 operational taxonomic units (OTUs), with genera including both common environmental fungi and taxa that contain members with pathogenic potential. Fungal assemblages differed markedly between air and feather samples. Insects, particularly Drosophilidae and Phoridae, carried spores both externally and internally, with notable detection of mycorrhizal *Thelephora aurantiotincta* spores in Blattodea digestive tracts. Our findings highlight the significant and distinct roles of both birds and insects in dispersing a diverse array of fungi, with potentially important implications for ecosystem processes, public health and forestry.

## Graphical abstract

Birds, insects and air each host distinct fungal spore communities in a Japanese temperate forest; birds and insects act as selective vectors, while air provides maximal diversity. Our study highlights the importance of analysing multiple taxa to understand ecosystem‐level fungal spore dispersal and interactions.

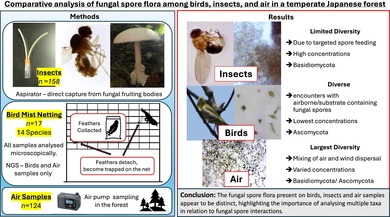

## 1 Introduction

Fungi are ubiquitous and highly influential as they provide multiple ecosystem services including nutrient cycling, decomposition, carbon sequestration, mutualistic associations with up to 90% of all plants (Lanfranco et al. 2016) and biodiversity support as they provide food and shelter for a diverse range of organisms (Bangay et al. 2022). Fungi have adapted to a multitude of environments as well as evolving various physical forms, but in order to colonise areas they require effective modes of spore dispersal (Roper et al. 2010).

The methods by which fungal spores are released into the environment have been extensively explored and are predominantly ejected from the fruiting body in two distinct ways, actively or passively (Chaudhary et al. 2022; Ingold 1971; Money 2016), utilising both abiotic and biotic vectors. The main abiotic vector of spore dispersal is wind (anemophily). Wind dispersal allows fungal spores to be carried thousands of kilometres where various plant pathogens have been suggested to remain viable after travelling across continents (Golan and Pringle 2017).

With the exception of plant pathogens and disease‐causing fungi, which can be tracked because their impact on human and animal health or crops is directly observable, monitoring the movement of most fungal spores, either directly or indirectly, is expected to require significant effort and specialised methodologies. This is due to the lack of visible, immediate effects for non‐pathogenic taxa and the technical challenges in detecting and tracking their dispersal through environmental sampling, biosensors or real‐time imaging approaches. Wind directions can change drastically and thus the final destination of fungal spores is extremely variable. Thus, little is known about their movement patterns across a range of spatiotemporal scales or their subsequent rate of successful germination, with an increasing reliance on predictive models (Grinn‐Gofroń et al. 2016).

Although wind‐borne spores require release from the fruiting body, the detachment of spores from the fruiting body is not necessary for animal‐vectored spores. Spores including those that have not been released from the fruiting body have been reported to be dispersed by animals through feeding. It has been reported that various mycophagous animals such as microarthropods, insects, mammals, reptiles and birds can disperse viable spores after passing through the digestive tract (Fagerli Lunde et al. 2023; Kobayashi et al. 2017; Tuno 1998). Depending on the vector, spore dispersal can occur across a range of distances (Chaudhary et al. 2022). For fungal species which are host specific or suited to specific habitats, that is, mycorrhizae, the employment of organismal vectors provides important implications and benefits, such as deposition into suitable environments for fungal germination. Biotic vectors can also carry spores externally, as is often the case with insects which contain multiple tiny setae (hairs) along their bodies. This allows for spores to become easily attached or embedded within them (Fagerli Lunde et al. 2023), this phenomenon has also been reported in mammals and birds (Johansson et al. 2025).

Non‐mycophagous animals may act as potential vectors of fungal spores, enabling modes of dispersal that differ from both airborne transmission and transport by mycophagous animals. In contrast to mycophagous species, which are known to carry spores internally and externally through feeding, evidence for spore dispersal by non‐mycophagous animals remains limited.

The main objective of this study is to elucidate the characteristics of both biotic and abiotic vectors of fungi by microscopy/molecular analysis and comparing fungal community composition from three sources: airborne spores in forests, fungi isolated from mycophagous insects and fungi isolated from non‐mycophagous forest dwelling birds. Understanding the spatial dynamics of fungal communities, which underpin key forest ecosystem functions, is essential for elucidating the mechanisms of fungal population regeneration and distributional expansion.

## 2 Materials and Methods

### 2.1 Study Sites

For feather collection, two forests were utilised. The vegetation of both forests, Kakuma (36°32′49.5″ N 136°42′18.9″ E: altitude: ~85 m above sea level) and Fukui (35°58′39.6″ N 136°01′08.6″ E: altitude: ~240 m ASL), consisted predominantly of Oaks *Quercus variabilis* , *Quercus serrata* , *Cryptomeria japonica* (Japanese Cedar) and *Phyllostachys edulis* (Bamboo) including a range of mixed deciduous trees and shrubs. The location at Fukui (Otayama Bird Handling Station) is in a mountainous region and is a prominent bird banding station.

### 2.2 Insect Capture

Insects were collected during the summer and autumn of 2024 within Kakuma Forest, Kanazawa University, to coincide with peak fungal fruiting season. Insects were predominantly collected from the mixed deciduous (MD) forest region as this is where a majority of our target fungi were located. An aspirator was used to collect insects directly from fungal fruiting bodies; to minimise cross contamination, separate collection containers were used for each fungus sampled for insects. The suction tubes were also wiped with an ethanol‐sprayed rod to remove particles from within the tube. Specific fungi were targeted for insect collection, particularly *Russula* and *Amanita* as these species are commonly frequented by mycophagous insects. On occasion, insects found on other fungal fruiting bodies were also collected. Insects were placed into a freezer for 20 min at a temperature of −20°C and then placed within 70% ethanol for preservation until further analysis.

### 2.3 Bird Capture and Feather Collection

Bird collection occurred on four different days. Bird capture was performed using a mist net (height 2.6 m: length 4–8 m) which occurred in the early morning during dry days until late morning. In Kakuma campus, the call of the Meadow Bunting ( *Emberiza cioides* ) was played beside the net to attract birds towards the net. Upon approach, the birds would become entangled within the net, allowing for handling and inspection. It is common for feathers to detach from birds during this process; therefore, only the detached feathers which were embedded within the mist net were collected and then placed into sealable plastic bags with the use of prewashed forceps. Feathers that had fallen to the ground were not collected to avoid contamination. The types of feathers collected from the birds were not discriminated or separately identified in terms of their positioning on the bird. In Fukui, due to an abundance of birds being captured, feathers were not separated by individuals but separated by species; therefore, multiple individuals from the same species were pooled into one feather collection. The three bird species collected from Kanazawa all came from specific individuals. Dates for bird collection can be found in Appendix S1: Table S2. Ethical approval was not required as no animals were handled or manipulated by the authors; all feathers were collected passively from mist nets by licensed handlers.

### 2.4 Forest Air Sampling

Air samples were collected using six standard air sampling pumps (MINIPUMP MP‐Sigma500NII; SIBATA Scientific Technology Holdings Ltd., Saitama, Japan) in threelocations (an open area, MD forest and bamboo forest) within Kakuma Forest, Kanazawa. Air samples were collected from between 12th June 2024–9th August 2024. Samples were collected at specific times of day and night: 11 pm–3 am, midday: 10 am–2 pm and 4 h from sunset. Pumps were placed at ground level and below canopy, and samples were collected simultaneously. The pump filtered air at a rate of 5 L/min, and fungal spores were captured onto a 25 mm glass fibre GF/D filter with a pore size of 2.7 μM (Watman Co. Ltd., Maidstone, UK).

### 2.5 Spore Analysis

#### 2.5.1 Insects

Insects were initially analysed microscopically at a range of magnifications between 100× and 400×. The exoskeletons were analysed for the presence of spores before being dissected. To observe the digestive tract, a couple of drops of 70% ethanol were placed onto the insect and then pressed with a cover glass to flatten the specimen and release any contents from the digestive tract. Each individual was photographed using a camera attachment (Swiftcam SC503; Swift Microscope World, Carlsbad, California, USA), as were any spores, and saved as jpeg or TIFF files using Swift Imaging 3.0 software. During sampling collection, small tissue samples from the gills of the fungi were also collected. Comparisons were made between the spores from the gills and spores located within the digestive tract of the insects. A reference fungal identification book (Ikeda 2013), which is specific to the area of Hokuriku (i.e., Fukui, Ishikawa and Toyama), was also used to cross‐reference spore morphology.

#### 2.5.2 Bird Feathers

Feathers from each sample were placed into separate prewashed 15 mL conical centrifuge tubes and submerged in 10 mL of ultrapure water (Milli‐Q; Merck Millipore, Massachusetts, USA), each tube was then vortexed (Vortex Genie 2; Scientific Industries Inc., New York, USA) continuously for 60 s, this was to dislodge spores present on the feathers. The water was then collected with a syringe and filtered through a 25 mm GF filter. The entire filter was then analysed microscopically for the presence of spores at a magnification of 200×–400×. Spores found were photographed and saved as jpeg or TIFF files. It should be noted that the number of feathers used differed between samples, as only detached feathers were collected, this varied between the samples. Three negative controls following the same protocol were also created to account for possible contamination during the water filtering/spore disruption process.

### 2.6 DNA Extraction From Filters

All feather, air samples and negative controls were prepared for DNA extraction. Initially, within a laminar flow hood which had been pretreated with ultraviolet light for 1 h, the filters were cut into very small pieces (approx. 1–2 mm) with prewashed (2% NaClO and distilled water) surgical scissors inside a 2 mL Eppendorf tube. To break the cell walls of the fungal spores, the cut filter was then submerged in 100 μL of 2× CTAB with two 5 mm zirconia beads and agitated using a Shake Master Neo (BMS Inc., Tokyo, Japan) at a rate of 1500 rpm for 10 min in total (5 min × 2). Phenol/chloroform was used for DNA extraction. The DNA pellet was purified using 70% ethanol precipitation and air dried overnight within a sterilised (wiped with 2% NaClO), non‐running laminar flow hood. The DNA pellet was dissolved in 50 μL of TE buffer and stored at −20°C.

#### 2.6.1 DNA Amplification and Library Preparation

The fungal internal transcribed spacer 1 region (ITS1) was amplified using KOD FX NEO using the following primers ITS1‐F‐KYO2 (5′‐TAG AGG AAG TAA AAG TCG TAA‐3′) and ITS2‐KYO2 (5′‐TAG AGG AAG TAA AAG TCG TAA‐3′) following Matsuoka et al. (2019). The master mix for one sample was as follows: KOD FX NEO Buffer 9.27 μL, dNTPs 3.7 μL, KOD FX NEO 0.36 μL, 5 μM forward primer 1.08 μL, 5 μM reverse primer 1.08 μL, distilled water 1.55 μL and DNA 1.55 μL, totalling 18.59 μL. The first polymerase chain reaction (PCR) protocol was as follows: an initial incubation for 2 min at 94°C, followed by 35 cycles of 10 s at 98°C, annealing 30 s at 54°C, 30 s at 68°C, with a final extension of 5 min at 68°C. Two PCR blanks consisting of PCR‐grade water were created to account for possible contamination during the amplification and library preparation process.

A second PCR using 2× KAPA HiFi HotStart ReadyMix (KAPA Biosystems, Wilmington, Washington, USA) was carried out with the addition of unique adapter sequencing indexes to each amplified sample. The master mix for one sample using KAPA HiFi was as follows: 2× KAPA HiFi HotStart ReadyMix 6 μL, each primer (2.5 μM) 1.4 μL and DNA template 3 μL, totalling 11.8 μL. The second PCR protocol was as follows: an initial incubation for 3 min at 95°C, followed by 12 cycles of 20 s at 98°C and annealing for 15 s at 72°C, with a final extension of 5 min at 72°C. Confirmation of successful amplification of the PCR products was carried out through electrophoresis on a 1.5% agarose gel, with 3 μL of PCR product combined with 0.6 μL of dye. Electrophoresis was carried out for 15 min at 100 v. After confirmation of DNA amplification, 2 μL from every sample was pooled into a single 2 mL low bind Eppendorf tube. Paired‐end sequencing was performed on an Illumina MiSeq platform.

### 2.7 Bioinformatics

For the raw FASTQ files, paired‐end reads were merged using commands implemented in the Claident pipeline (Tanabe and Toju 2013; software available online, http://www.claident.org/). Potentially noisy and chimeric sequences were eliminated using DADA2 (Callahan et al. 2016). Remaining reads were then screened again for chimeric sequences which were removed using UCHIME (Edgar et al. 2011) through both reference‐based (using UNITE database) and de novo detection methods to ensure comprehensive chimera removal. After these procedures, reads were clustered into Operational Taxonomic Units (OTUs) at a 97% similarity threshold using the ‘clclassseqv’ command. Taxonomic classification was performed using two complementary approaches. The RDP Naive Bayesian Classifier, implemented in the DADA2 pipeline (Wang et al. 2007), was used to assign OTUs to genus and higher taxonomic ranks. Although RDP can provide species‐level assignments, its accuracy at finer taxonomic resolution in ITS datasets can be limited by database coverage. Therefore, species‐level identifications were refined using the Claident QCauto LCA algorithm, which applies similarity‐based assignments with stringent thresholds and yields fewer but higher‐confidence species names.

In summary, RDP provides broad and relatively inclusive taxonomic coverage, which is useful for describing overall community patterns at genus and higher ranks, whereas Claident QCauto–LCA yields fewer but more conservative and reliable species‐level assignments. Both methods are presented because RDP classifications were used for community‐level summaries and ordination analyses, while Claident assignments were used when high‐confidence species‐level information was required.

All abundance tables were normalised using cumulative sum scaling (CSS) via the microeco package. CSS normalisation was chosen because it corrects for sequencing depth differences and reduces the influence of highly abundant features, providing robust sample comparisons and controlling for library size biases in fungal community sequencing data (Paulson et al. 2013).

### 2.8 Statistical Analyses

#### 2.8.1 Fungal Spore Flora

The R package ‘microeco’ version 1.15.0 (Liu et al. 2020) was utilised to analyse the fungal spore flora data as the package contains multiple applicable methods of analysing microbial data, with access to a range of diversity indices and statistical tests. Statistical tests and ordination analyses included non‐metric multidimensional scaling (NMDS), principal coordinate analysis (PCoA) and canonical correspondence analysis (CCA). Principal coordinates analysis (PCoA) was conducted to explore the differences in fungal spore flora among bird samples collected from Kanazawa and Fukui (Appendix S1: Figure S9). Detrended correspondence analysis (DCA) indicated unimodal species responses (gradient length > 4 SD units), so CCA was used as the primary constrained ordination. NMDS and PCoA were applied as complementary unconstrained visualisations. Community composition at the OTU level was analysed using PERMANOVA and ANOSIM based on Bray–Curtis dissimilarities. All samples collected in Kanazawa were during summer, whereas all samples from Fukui were collected during autumn; therefore, specific influences from area and season cannot be separated and distinguished. All species‐level analyses are based on Claident taxonomic output, whereas higher classifications are analysed using RDP taxonomic output.

#### 2.8.2 Ecological Trait Assignment and Analysis

To investigate the ecological functions of fungal spore flora across bird species, OTUs were analysed using the FUNGuild database. Taxonomic assignments were matched to ecological guilds (i.e., saprotroph, plant pathogen, endophyte) and trophic modes based on trait information. The results were then visualised using heatmaps to outline differences in ecological function across the samples.

#### 2.8.3 Comparison of Air Spore Flora vs. Bird Spore Flora

To compare the airborne fungal spore flora against the fungal spore flora found on bird feathers, air samples collected from Kakuma forest, relating to the same time that bird samples were collected, were used. Air samples consisted of 124 samples collected between 12th June 2024–9th August 2024. An NMDS was used to visualise groupings of fungi and was further analysed through detrended correspondence analysis (DCA) (Appendix S1: Figure S10).

## 3 Results

### 3.1 Insects

Overall, 158 invertebrates were collected across 12 days from June 2024 to November 2024. A majority of the insects collected were from the mixed deciduous forest location (*n* = 140), followed by a less vegetative open area within the same forest (*n* = 18). Insects were collected from 11 different fungal families consisting of 15 identified species. From the 158 insects, 72 (45.6%) were found to carry fungal spores either internally or externally (Table 1), two invertebrate specimens were removed due to an inability to accurately identify them; neither carried spores.

**TABLE 1:** Insects collected from fungi using an aspirator. Displays the percentage (%) of insect individuals of which fungal spores were found, either internally or externally.

Fungal specimen	Insect family	No. specimens	With spores	% With spores	Spore location	Host/non‐host
*Amanita eijii*	Culicidae	1	0	0	—	—
—	Scaphididae	2	0	0	—	—
—	Staphylinidae	5	0	0	—	—
*Amanita spissacea*	Culicidae	1	0	0	—	—
—	Drosophilidae	7	4	57.1	Digestive tract, ext.	Non‐host
—	Formicidae	5	1	20	Digestive tract	Non‐host
—	Phalacridae	10	0	0	—	—
—	Scaphididae	4	0	0	—	—
—	Sciaridae	1	0	0	—	—
—	Staphylinidae	2	0	0	—	—
*Amanita virgineoides*	Phoridae	5	3	60	Leg joints, thorax, abdomen	Non‐host
—	Sciaridae	1	1	100	Wing, internal	Non‐host
*Amanita virosa*	Diapriidae	1	0	0	—	—
—	Drosophilidae	1	1	100	Digestive tract	Non‐host
—	Formicidae	1	1	100	Mandibles (external)	Non‐host
—	Morphospecies 2	3	0	0	—	—
—	Staphylinidae	4	2	50	Thorax (external)	Non‐host
*Clitocybe nebularis*	Diapriidae	1	0	0	—	—
—	Heleomyzidae	6	5	83.3	Digestive tract, thorax	Host + non‐host
—	Morphospecies 3	2	0	0	—	—
—	Morphospecies 4	1	0	0	—	—
—	Phoridae	2	0	0	—	—
—	Sciaridae	1	0	0	—	—
*Flammulina velutipes*	Staphylinidae	16	14	87.5	Digestive tract	Host
*Inonotus mikadoi*	Drosophilidae	2	1	50	Digestive tract	Host
—	Formicidae	1	1	100	Head, thorax (external)	Host
—	Staphylinidae	1	0	0	—	—
*Laetiporus sulphureus*	Phalacridae	3	0	0	—	—
*Mycena galericulata*	Drosophilidae	5	5	100	Digestive tract, thorax	Host
—	Phalacridae	1	1	100	Digestive tract	Host
—	Phoridae	1	0	0	—	—
*Panaeolus papilionaceus*	Phalacridae	1	1	100	Digestive tract	Host
*Pleurotus ostreatus*	Scaphididae	6	1	16.7	Under elytra (external)	Non‐host
*Russula* sp.	Cecidomyiidae	1	0	0	—	—
—	Diapriidae	1	0	0	—	—
—	Drosophilidae	22	15	68.2	Digestive tract, legs (external)	Non‐host
—	Formicidae	2	0	0	—	—
—	Heleomyzidae	2	2	100	Digestive tract, thorax	Non‐host
—	Mycetophilidae	1	0	0	—	—
—	Phoridae	2	1	50	Digestive tract	Non‐host
—	Sciaridae	1	1	100	Legs (external)	Non‐host
—	Trichoceridae	1	1	100	Wings, abdomen	Non‐host
*Russula virescens*	Drosophilidae	2	2	100	Digestive tract	Non‐host
*Thelephora aurantiotincta*	Blattodea Morphospecies 1	2	2	100	Digestive tract	Host
—	Scaphididae	2	0	0	—	—
—	Staphylinidae	3	0	0	—	—
*Tyromyces chioneus*	Drosophilidae	9	6	66.7	Digestive tract, thorax	Non‐host
—	Scaphididae	1	0	0	—	—
	Total	156	72			

**FIGURE 1:**
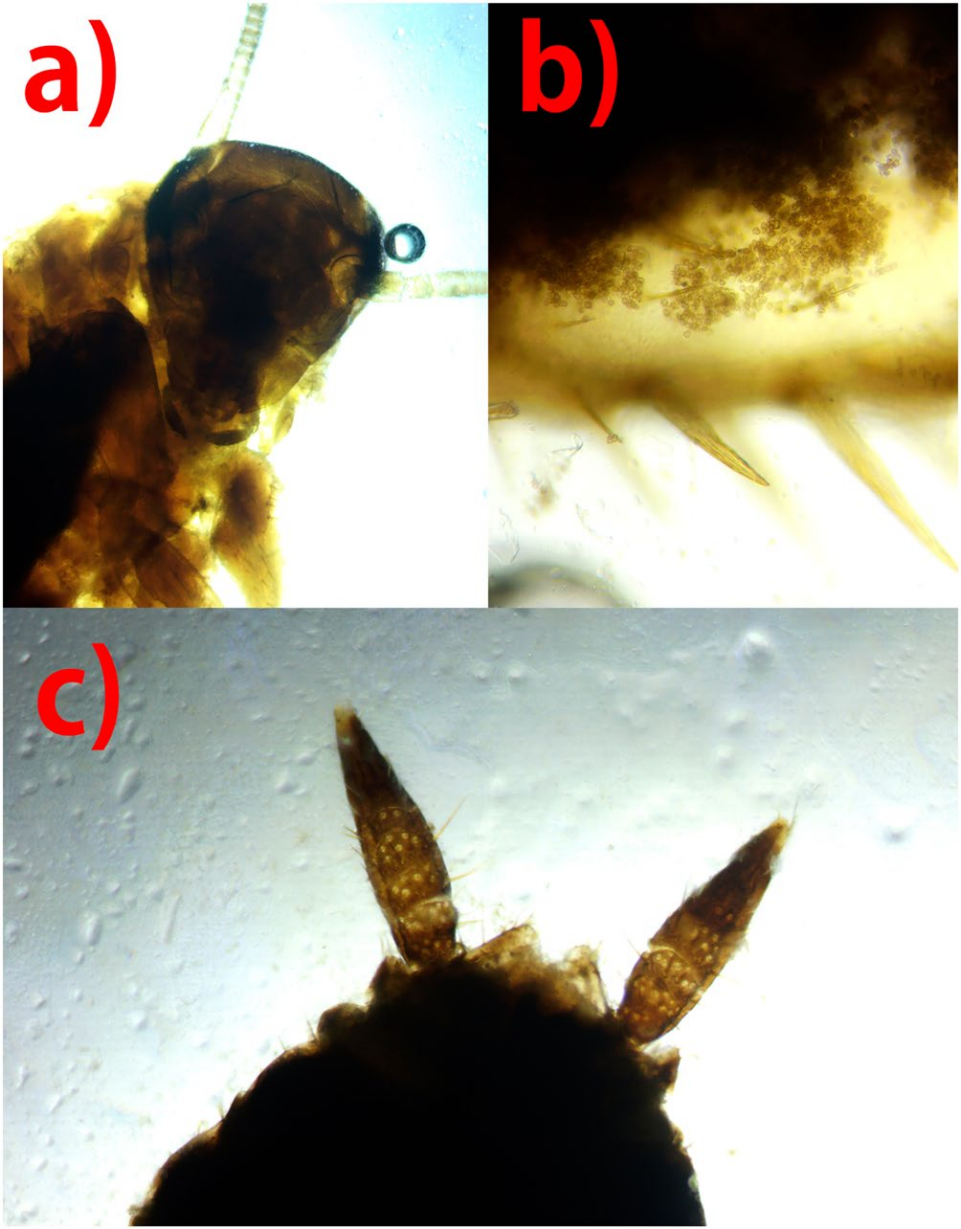
A member of the order Blattodea (Cockroach), carrying fungal spores belonging to the mycorrhizal *Thelephora aurantiotincta*. (a) Head, (b) fungal spores from within the digestive tract, (c) Cerci, appendages found at the terminal end of the cockroach.

Many of the spores being carried by insects did not match the spores of the fungi from which they were collected (Table 1). Drosophilidae and Staphylinidae were the most abundant insects to be collected from fungal fruiting bodies, 47 and 30 individuals, respectively. Over 70% of Drosophilids contained fungal spores and over 50% of Staphylinids contained fungal spores (Table 2). Fourteen Staphylinids which contained fungal spores within their digestive tracts were in their larval stage. Predatory species such as Formicidae also contained fungal spores (Figure 1).

**TABLE 2:** Insect family and the overall percentage of individuals which contained spores either internally or externally.

Family	Examined	With spores	% With spores
Drosophilidae	47	33	70.2
Staphylinidae	30	16	53.3
Scaphididae	15	1	6.7
Phalacridae	15	2	13.3
Formicidae	8	3	37.5
Heleomyzidae	8	7	87.5
Phoridae	10	4	40
Sciaridae	4	2	50
Blattodea	2	2	100
Diapriidae	3	0	0
Culicidae	3	0	0
Mycetophilidae	1	0	0
Cecidomyiidae	1	0	0
Trichoceridae	1	1	100
Others/unidentified	8	1	10
Total	156	72	

### 3.2 Birds

Overall, across four separate days of mist netting, the feathers from 17 samples consisting of 14 bird species were collected, three species from Kakuma Campus, Kanazawa, Japan, and 11 from Fukui, Japan. Various species from the genus *Turdus*, that is, thrushes, were the most abundant, followed by buntings and warblers.

### 3.3 Fungal Spore Flora: Feather Samples

Fungal DNA was successfully extracted from feathers from all 14 bird species. Three negative controls and two PCR blanks included in each sequencing run yielded a total below 100 reads, indicating the absence of significant contamination in the workflow. The total number of raw reads from 17 samples was 919,787 consisting of 479 operational taxonomic units (OTUs). A baseline threshold of minimum reads per OTU (10 reads) was set using ‘microeco’ and after removal of low read OTUs, abundance tables were then normalised via cumulative sum scaling (CSS). Post‐filtering and normalisation, the dataset comprised 405 OTUs corresponding to 196 assigned fungal species (based on RDP classification output). For Claident output however, raw data after post filtering and normalisation resulted in 381 OTUs consisting of 39 assigned fungal species.

**FIGURE 2:**
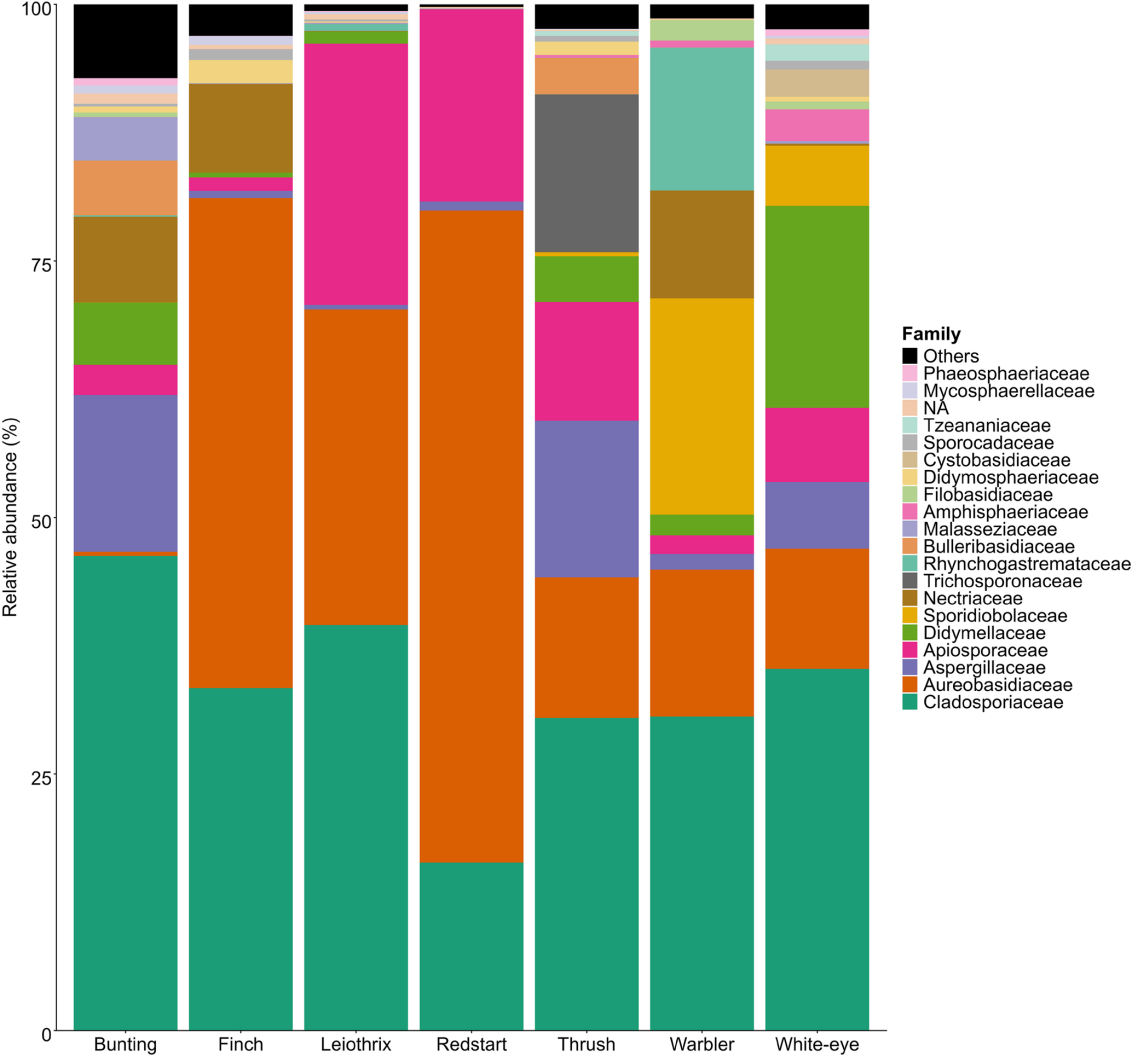
Stacked barplot showing the relative abundance of fungal families associated with each bird group. Each bar represents a bird grouping, with different coloured segments indicating the proportional abundance of each detected fungal family.

In terms of taxonomy and relative abundance, at phylum level, Ascomycota consisted of 197 OTU's (78.5%) and Basidiomycota consisted of 52 OTU's (20.7%), 2 OTU's were unassigned to a phylum. Of the Ascomycota, 24 orders were identified, the most common being Pleosporales (62 OTU's), Capnodiales (24 OTU's), Hypocreales (21 OTU's), Xylariales (20 OTU's), Eurotiales (19 OTU's) and Basidiomycota consisted of 19 orders, most common being Tremellales (11 OTU's), Polyporales (10 OTU's), Malasseziales (7 OTU's), Filobasidiales (3 OTU's). The most numerous Ascomycete genera were *Aspergillus* (9 OTU's), *Penicillium* (9 OTU's) and *Arthrinium* (8 OTU's). For Basidiomycetes, the most numerous genera were *Malassezia* (6 OTU's), *Dioszegia* (3 OTU's) and *Papiliotrema* (3 OTU's). Dominant families include Cladosporiaceae, Aureobasidiaceae and Aspergillaceae across host species (Figure 2).

**FIGURE 3:**
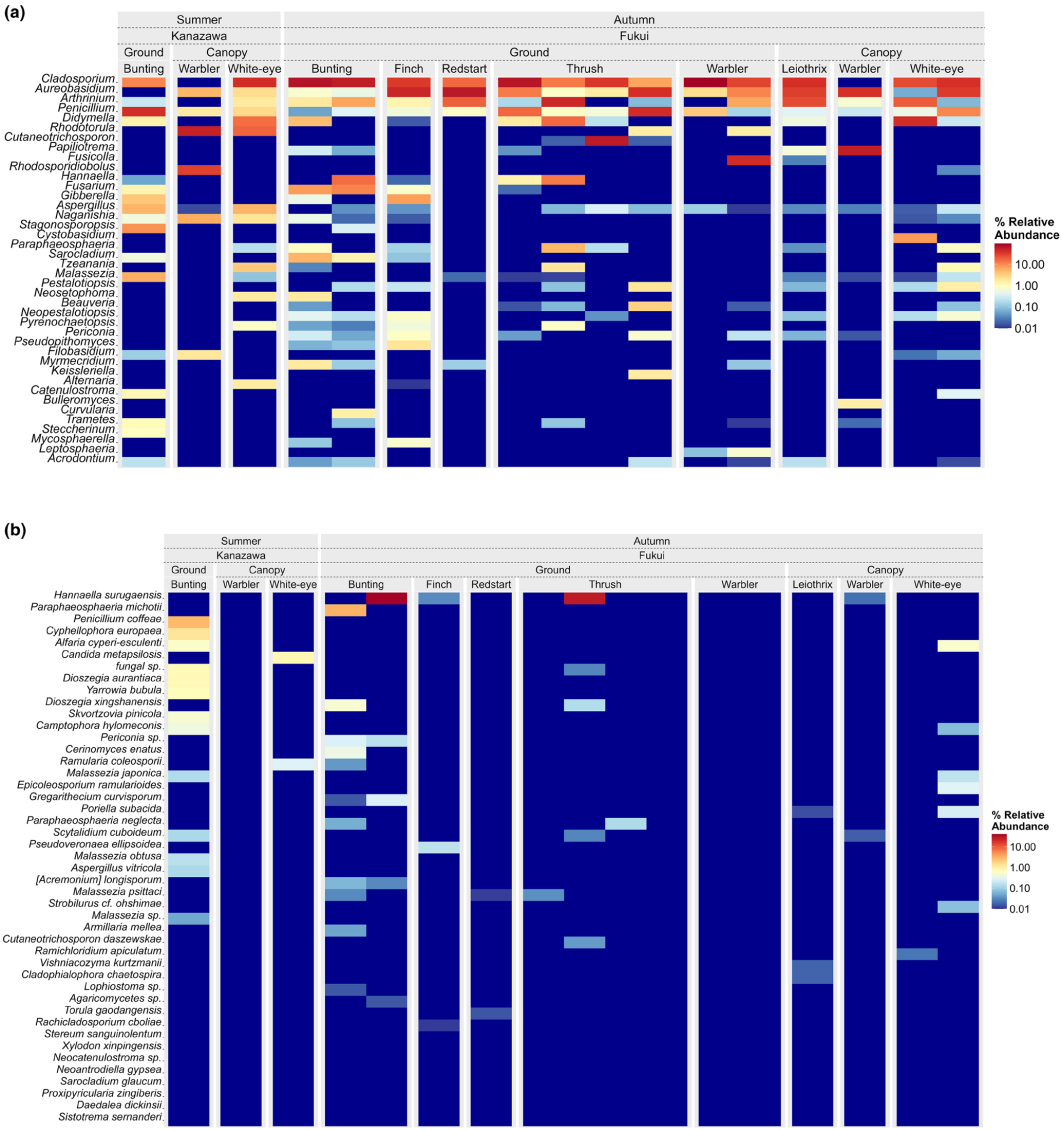
(a) Heatmap of fungal genus relative abundance across bird foraging guilds and sampling areas. Columns represent combinations of foraging behaviour (ground vs. canopy), bird types and geographical areas (Kanazawa and Fukui), while rows indicate detected fungal genera. Colour intensity depicts percent relative abundance on a log scale. Kanazawa and Fukui are shown side‐by‐side for each guild comparison, highlighting differences in fungal community composition among habitats and bird types. (b) Heatmap of fungal species relative abundance across bird foraging guilds and sampling areas.

Fourteen birds harboured a higher relative abundance of Ascomycota, whereas three birds harboured more Basidiomycetes (2 warblers and 1 thrush) (Appendix S1: Figure S6). At genus level, *Penicillium* was present in all 17 bird samples (Figure 3a). Fungal genera *Cladosporium*, *Aureobasidium*, *Arthrinium*, *Penicillium* and *Didymella* were also highly abundant (Figure 3a). At species level, a range of fungi were assigned (Figure 3b). Pielou's evenness index was calculated to compare fungal spore flora evenness between areas (Kanazawa and Fukui) using the Wilcoxon rank sum test. The analysis reveals a significant difference in evenness between the two areas (*p* = 0.02), with Kanazawa showing a higher mean evenness (mean = 0.63, SD = 0.03) than Fukui (mean = 0.4, SD = 0.12).

**FIGURE 4:**
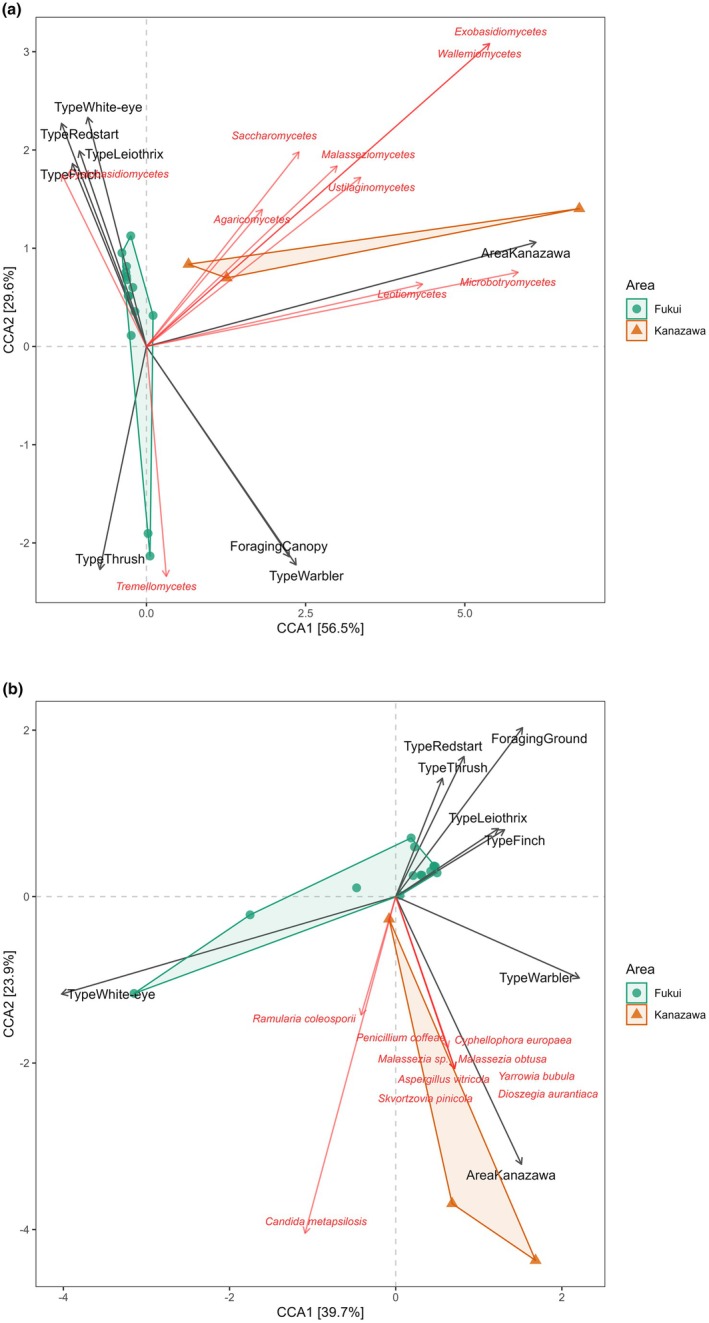
(a) Canonical correspondence analysis (CCA) biplot illustrating the relationship between fungal class composition and bird foraging guilds across two sampling areas (Fukui: green circles; Kanazawa: orange circles). Points represent individual samples coloured by area, with black arrows indicating environmental variables (bird types and sampling area) and red arrows representing fungal classes. The length and direction of arrows indicate the strength and association of each variable with the ordination axes. (b) Canonical correspondence analysis (CCA) of fungal spore flora (taxonomic level: species) in association with bird genera, location and foraging behaviour.

Canonical correspondence analysis (CCA) revealed that fungal spore flora at class level was correlated with multiple variables, namely area, bird grouping and foraging behaviour (ground vs. canopy). Overall, 63.7% of the entire variation in fungal spore flora was explained by area/season, foraging and bird grouping; 36.3% is unaccounted for. Of the 63.7%, axis 1 (area/season) and axis 2 (relating to bird grouping and foraging behaviour) explain 86.1% of the constrained variance when considering fungal class. Tremellomycetes were clustered with ground‐foraging Thrushes within Fukui whereas Agaricomycetes and Leotiomycetes were more abundant in Kanazawa/summer samples (Figure 4a). This distinction between the two sample types is further illustrated by detrended correspondence analysis (DCA), which reveals clear separation in community composition between air and feather samples (Figure 6).

Canonical correspondence analysis at the species level revealed that 57.6% of the total variation in feather‐associated fungal spore community composition was explained by area/season, bird foraging strategy and bird grouping, with 42.4% of variation remaining unaccounted for. The first two canonical axes accounted for 39.7% and 23.9% of the constrained variation, respectively. A significant effect of area on community structure was detected (PERMANOVA: *F* = 1.69, *p* = 0.035), and ANOSIM indicated moderate but significant separation between Fukui and Kanazawa (*R* = 0.39, *p* = 0.024). There is distinct clustering of Fukui samples with ground‐foraging birds (Thrushes, Redstart), while Kanazawa samples and canopy foragers were associated with a different subset of fungal species, including *Penicillium coffeae*, *Malassezia* sp. and *Dioszegia aurantiaca* (Figure 4b).

### 3.4 Fungal Spore Flora: Feather Samples vs. Forest Air Samples

To compare fungal communities between spores collected from bird feathers collected in Kanazawa (*n* = 3) and air samples within a nearby forest during the same time period (*n* = 124), DNA from a total of 124 air samples was extracted and sequenced. The total number of raw reads from all 127 samples was 1,525,561 consisting of 5619 OTUs. After post‐filtering and normalisation, the dataset comprised 2736 OTUs corresponding to 859 assigned fungal species (based on RDP classification output). For Claident output however, raw data after post filtering and normalisation resulted in 2634 OTUs consisting of 244 assigned fungal species. Species‐level assignment of fungi analysed on bird samples was identical for both RDP and Claident.

**FIGURE 5:**
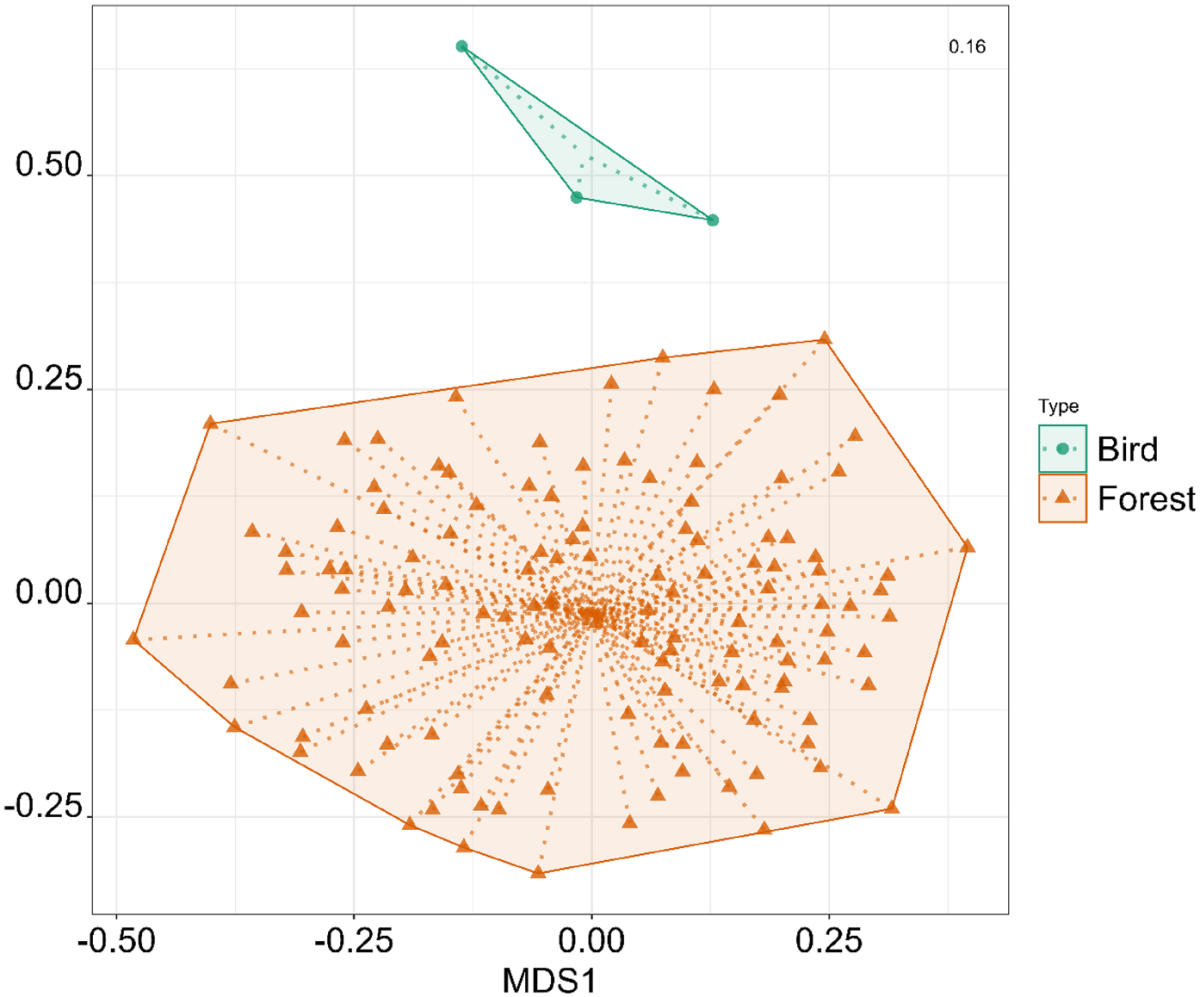
Non‐metric multidimensional scaling (NMDS) ordination plot comparing fungal community structure between bird (*n* = 3) and forest air samples (*n* = 124) in Kanazawa. Points represent individual samples, grouped and enclosed by polygons according to sample type (bird: circles and green polygon; forest: triangles and orange polygon). The spread and separation of the polygons illustrate differences in community composition between sample types, with birds showing greater dispersion and distinct clustering compared to forest samples. Stress value (top right) indicates ordination fit quality.

**FIGURE 6:**
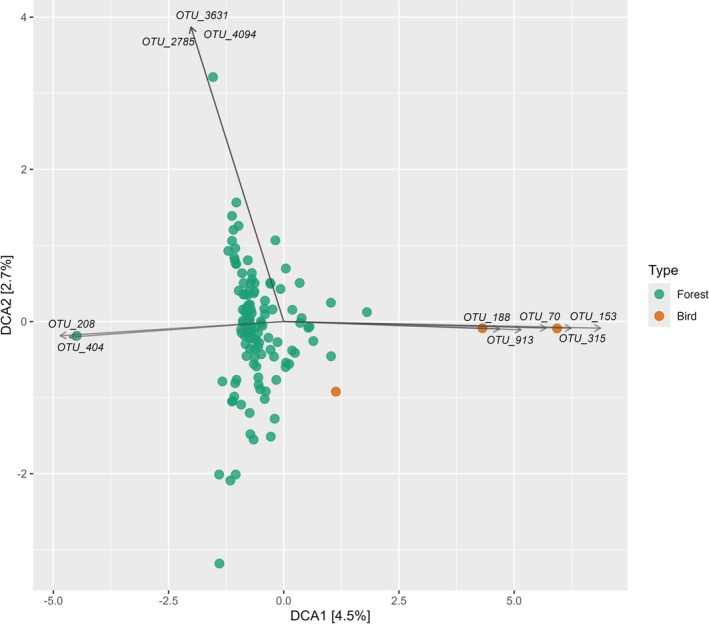
Detrended correspondence analysis (DCA) biplot illustrating compositional differences between fungal OTUs recovered from forest air samples (green circles) and bird‐associated samples (orange circles). Axes represent the first two DCA components (DCA1: 4.5%, DCA2: 2.7% of total variance). Selected OTUs contributing most strongly to variation along DCA1 and DCA2 are indicated by arrows and labels. The ordination highlights the separation between community structures typical of forest environments and those detected in association with birds.

The fungal spore flora identified on bird feathers collected within Kanazawa differed significantly from the fungal spore flora collected from air samples in Kakuma Forest between June and August (PERMANOVA, *F* = 2.72, *p* < 0.001; Figure 5). Several OTUs were strongly associated with bird feather samples, including OTU_153 (Sporidioborales), OTU_913 (Boletales), OTU_70 (unassigned), OTU_315 (unassigned) and OTU_188 (unassigned). In contrast, OTU_208 (Dothideomycetes), OTU_404 (Eurotiomycetes), OTU_2785 (Hemimycena), OTU_3631 (Tilletiaceae) and OTU_4094 (unassigned) were linked more closely with forest air samples (Figure 6).

**FIGURE 7:**
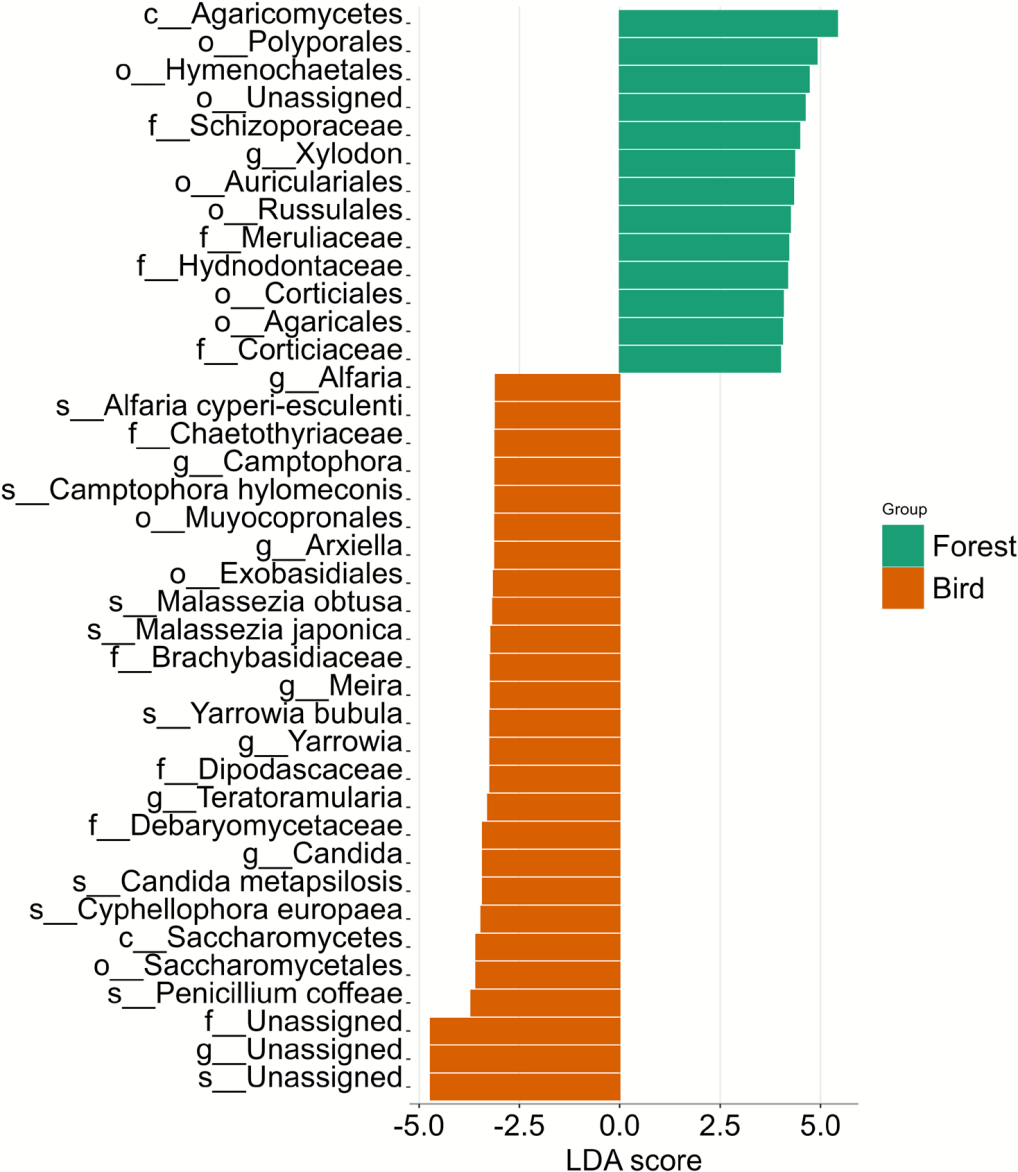
Linear discriminant analysis (LDA) scores for fungal taxa differentially associated with forest air samples (green) and bird feather samples (orange). Taxa are listed on the *y*‐axis at the highest identified rank (class, order, family, genus, or species using Claident). Positive LDA scores indicate taxa associated strongly with forest samples, while negative scores correspond to taxa associated with bird feathers.

From the 244 fungal species assigned by Claident, six species were found to be present only within bird samples (Table 3), which is also indicated by Linear discriminant analysis, which shows various species strongly associated with bird samples, specifically *Alfaria cyper‐esculenti*, *Camptophora hylomeconis*, *Malassezia species*, *Yarrowia bubula* among others (Figure 7).

**TABLE 3:** List of six fungal species identified through Claident collected from bird samples that were not present in 124 air samples analysed within Kanazawa. Species names were manually confirmed by BLAST against type or phylogenetically validated ITS sequences (NR_111474.1, NR_126117.1, NR_120101.1, NR_155999.1, LC811689.1, LC389043.1, NR_165186.1).

Species	Ecological role	Pathogenicity	Habitat/host
*Camptophora hylomeconis*	Saprotroph	Not reported as a pathogen	Decaying plant/leaf litter, wood
*Candida metapsilosis*	Commensal/opportunist	Opportunistic pathogen	Human mucosa, skin, marine, birds
*Cyphellophora europaea*	Environmental/Opportunist	Occasional skin/nail infections	Soil, decaying wood, skin, feathers
*Malassezia japonica*	Commensal/Opportunist	Skin pathogen possible	Skin (birds/humans)
*Malassezia obtusa*	Commensal	Rare skin pathogen	Mammalian skin, sebaceous areas
*Yarrowia bubula*	Environmental yeast	Non‐pathogenic	Soil, insects, feathers, foods

## 4 Discussion

We aimed to identify and compare the fungal spore flora associated with biotic and abiotic vectors within a forested habitat; in doing so it has been distinguished that a highly diverse range of fungal spore flora is present within the air and to a lesser extent upon the feathers of multiple bird species whereas insects are liable to carry highly concentrated numbers of few species of fungal spores internally. Externally, insect species are capable of vectoring a range of fungal spores at extremely low concentrations. Here we will discuss the implications of particular vectors transporting distinct spore flora as well as highlighting various taxa which may have important economic impacts due to possible pathogenic risks.

### 4.1 Insects

There are multiple species of Drosophila which are associated with fungi including *D. bizonata* and *D. angularis* , and multiple individuals were found to contain high concentrations of intact spores within their digestive tracts (Appendix S1: Figure S2). Often, spores observed within the digestive tract did not reflect the fungal species from which they were collected, highlighting the insects' ability to travel from one fungus to another. However, there were two fungal specimens (*Amanita eijii* and *Laetiporus sulphureus*) where no spores were present from the collected insects (Appendix S1: Table S1). Drosophilids often demonstrate crepuscular activity patterns, showing clear periods of activity during dusk and dawn (De and Chatterjee 2022), Basidiomycota, appear to preferentially release their spores at night (Lagomarsino Oneto et al. 2020), this may be pivotal in the success of spore dispersal as these fungi may utilise multiple vectors throughout the day and night (Kobayashi et al. 2017).

A predatory ant was also found to carry spores, externally, within joint spaces between appendages connecting the thorax and legs (Appendix S1: Figure S1a,1b), these small crevices provide a space for which spores can become embedded. It was common to find ants on multiple fungal species of *Amanita*, *Russula* and *Inonotus mikadoi* exhibiting their predatory foraging behaviour. Ant nests are commonly found underground within the soil and are likely to interact with multiple tree roots, allowing them to be a prime candidate for effective spore dispersal, especially for mycorrhizal fungi which are often host specific.

Fungal spores belonging to the genus *Pestalotiopsis* were also found to be present on a number of insect species, both internally and externally (Appendix S1: Figure S3a,b). As fungal fruiting bodies begin to deliquesce, many generalist saprophagous insects will be able to feed upon the fungal material, including fungal spores (Bangay et al. 2022); these saprophages may also end up transmitting plant pathogenic or saprophytic fungi to nearby plants as they move from one feeding material to another.

The finding of Blattodea carrying an abundant number of *Thelephora aurantiotincta* spores is a potentially important observation, and to our knowledge, there are no records globally which indicate Blattodea as spore vectors of a mycorrhizal fungus. The density of spores found within both individuals of Blattodea was visually the highest of all insects (Figure 1). The spores appeared to be intact and consistent with that of *T. aurantiotincta*. However, due to only two specimens being collected, more information and data are required to understand whether this is a true fungal‐insect interaction. There is evidence to suggest that the Japanese forest cockroach, *Blattella nipponica*, is capable of dispersing the seeds of the plant *Monotropastrum humile*, a fungal parasite of mycorrhizal fungi (Uehara and Sugiura 2017), and so the close association between fungi—plant—cockroach may also crossover to spore dispersal of mycorrhizal fungi by cockroaches.

Insects are highly abundant during the summer and autumn seasons in temperate Japanese forests, and due to their foraging behaviour and targeting of specific fungal species, insects can accumulate large concentrations of fungal spores. Additionally, through chance accumulations of fungal spores upon the exoskeletons, non‐targeted fungal species may be utilising both wind and insect vectors as dispersal methods.

### 4.2 Birds

Firstly, multiple fungal genera detected on bird feathers include members known to contain pathogenic species, including potential animal and plant pathogens as well as saprotrophs (Appendix S1: Figures S4, S5 and S11). It has been well documented that birds suffer from fungal infections, especially those relating to skin and feathers. Genera known to include species capable of causing Aspergillosis (*Aspergillus*), Candidiasis (*Candida*) and Rhodotoruliasis (*Rhodotorula*) were detected in bird feather samples (Nardoni and Mancianti 2021; Garcês 2023) (Figure 3). While these taxa include known human pathogens, our study did not assess the viability or transmissibility of these spores. Nonetheless, this highlights the need for caution and further research into potential zoonotic vectors in avian microbiomes, particularly for those who regularly handle wild or captive birds. Given the detection of fungal genera that include avian and potentially zoonotic pathogenic species in our samples, future research should evaluate the frequency and relevance of such transmissions in settings with close bird‐human contact, including the poultry industry and public recreational spaces.

Much of the fungal flora appears to be strongly associated with water. Various fungi can thrive in wet and humid sites including water bodies (i.e., rivers/lakes) (Matsuoka et al. 2019). The presence of such fungi may be reflective of the birds drinking and bathing behaviours. Passerine birds must stay well hydrated during summer months when temperatures are at their highest, and frequent visits to water bodies can be helpful for thermoregulation and removal of parasites (Bush and Clayton 2018). Lichen‐associated fungi were also present on several bird species, likely reflecting their foraging and perching habits in tree canopies which are commonly covered with lichen.

The importance of rain in fungal dispersal should not be ignored, especially in relation to birds; for example, bird movement or flight during heavy rain is drastically reduced. It is common for many birds to remain perched on branches within vegetation during heavy rain due to water retention on the feathers and reduced aerodynamics, resulting in higher energy consumption (Ortega‐Jimenez and Dudley 2012). Combined with the splash dispersal effect of rain and the high abundance of Ascomycota found below canopy, an area where many birds are prone to taking shelter, the common behavioural trait of flight avoidance during rainfall may greatly increase fungal spore transmission from branches or leaf surfaces to the bodies of birds.

The finding of mycorrhizal fungi upon bird feathers is important in relation to fungal dispersal because many passerine birds can often be territorial, and if excluded from a territory, are likely to find a new territory within a similar environment, thus the relationship between forests and many bird species can be tightly linked. Therefore, it would be beneficial for mycorrhizal fungi to be able to utilise birds as a fungal spore vector as the likelihood of spores being deposited into suitable areas is higher than relying on wind dispersal (Caiafa et al. 2021; Johansson et al. 2025). Additionally, according to RDP taxonomic assignment, the presence of wood rotting fungi such as *Bjerkandera adusta* and *Trametes versicolor* (both collected from feathers belonging to a Meadow Bunting ( *Emberiza cioides* )) may also reflect the ground‐foraging behaviour of this bird as these fungi along with others are commonly found close to the ground (Appendix S1: Figure S8) and also indicates that these fungi may utilise both wind and organismal vectors to disperse their spores.

Another key aspect of avian ecology which strongly links to seasonality is migration. During autumn and winter, multiple bird species undergo migrations across a range of distances. Species residing in mountainous regions during summer will migrate to lowland regions during autumn and winter due to drops in temperature at higher altitudes. The movement of birds between forests at varying elevations may prove to be extremely important for fungal spore dispersal, and potentially mycorrhizal colonisation between varying regions (Caiafa et al. 2021).

Overall, it appears that birds' natural behaviours and lifestyles may be partially reflected in the fungal spore flora of which they support (Johansson et al. 2025) with a number of the fungal species identified from this study correlating with previous findings regarding avian‐mycology interactions (Irga et al. 2016; Johansson et al. 2025).

### 4.3 Insects, Birds and Air

There appears to be interesting connections between atmospheric fungal spores and fungal spore flora on bird feathers as well as links between atmospheric fungal spores, fungal fruiting bodies and insects.

The fact that bird feathers carry a distinct fungal spore flora compared to airborne fungal spore flora collected from the same forest during the same period (Figure 5, Appendix S1: Figure S10) suggests that certain fungi may be resident on the bodies of birds, perhaps relating to skin or feather infections. However, the DNA analysis of the spores present on their feathers also indicates that fungal spores from multiple genera/species often found in the air, that is, *Cladosporium*, *Aspergillus vitricola* and *Penicillium coffeae*, are also prevalent across bird species. Moreover, given the overall distinctive spore flora assemblage found between air samples and bird feathers, tactile interactions with a range of substrates may be a more likely method of spore accumulation and cause between the differences in fungal spore flora between air and feather samples.

Furthermore, a majority of birds are omnivorous and will often consume insects throughout the year. The consumption of insects by birds may facilitate secondary spore dispersal however further research is required to validate this. The fact that *Pestalotiopsis* spores were found both on insects as well as being present and assigned as *P. parva* (RDP classification), a common plant pathogen, within the forest air samples may also indicate insects ability to capture airborne spores. Multiple ground‐based fungi such as *Amanita* and *Russula* were assigned to species level using both RDP and Claident and a number of insects were collected from *Amanita spissacea* where spores were present within air samples as well as within insect species, this further supports the possibility that fungi may utilise multiple methods to disperse their fungal spores into the surrounding environment.

### 4.4 Limitations

This study has several limitations that should be considered when interpreting the results. Firstly, it is difficult to distinguish the main driver of dissimilarity between fungal spore flora and area/season due to their confounding effects (Figures 3 and 4, Appendix S1: Figures S6 and S7) and limited sample size. Seasonality greatly influences fungal communities. With regards to this study, it seems more likely that the differences in fungal spore flora are due to seasonality, rather than area, as the dominant vegetation is similar across both sites, therefore it is to be expected that much of the ground‐based fungal communities are also likely to be similar, but overall, there is not enough evidence to infer with confidence the true driving factors between these differences. Furthermore, the low number of feather samples collected from Kanazawa (*n* = 3) hampers the reliability of the inference from these results and greatly limits the statistical power of analyses which prevents making any clear generalisations of fungal spore flora present on bird feathers and should therefore be considered as preliminary findings, however with such limited data currently available, it certainly warrants further research and provides an important basic framework for future studies.

In terms of spore collection and identification from the insects themselves, there is a risk that some spores may have been overlooked or perhaps lost during the transferal of specimens from the collection chamber to the microscope slide. There is also the possibility that some spores within the digestive tract may have been digested beyond recognition. Despite these limitations, all insect specimens were checked thoroughly at a range of magnifications providing important information and evidence of spore retention both externally and internally.

Despite careful washing and handling protocols, there remains an unavoidable risk of contamination during the feather collection and processing steps. The feather collection method requires further refinement to reduce the risk of contamination. The samples collected at Fukui consisted of many individuals, and for some species, feathers from multiple individuals of the same species were pooled into one sample, obscuring inter‐individual variation, reducing the resolution of our results regarding host‐specificity and intra‐population differences in fungal spore flora. However, this does not detract from the main objectives of investigating the fungal spore flora present on bird feathers, but does limit the fine scale data associated with variation between individuals.

Additionally, having filters exposed to the air during feather collection may be an effective way to assess contamination risks in future studies. Lastly, the number of feathers used to analyse fungal spore flora differed between samples as the number of detached feathers from the birds varied, which may have caused differences between fungal detection. To combat this in the future it may be worthwhile to standardise the size of feathers to be used as well as aiming to collect feathers from specific parts of the body, that is, primaries or tailfeathers. Nevertheless, the fungi that were present on the bird feathers within this study appear to closely resemble fungi found on bird samples in the literature.

Despite these limitations, the current study provides foundational insights and establishes a framework for further investigations into the ecological dynamics of fungal spore dispersal by birds and insect vectors.

### 4.5 Future Research

This study has showcased how feathers are capable of carrying a large array of fungal flora and attempts have been made to compare ground‐foraging versus canopy foraging. To truly explore this, it would be most effective to sample areas which contain birds that portray clear distinctions between foraging behaviours, that is, flightless versus flying birds; this would be a more robust approach to comparing foraging habits of birds to the possible fungal flora they may carry. A suitable area to investigate such interactions would be the temperate forests of New Zealand, a country which is home to the largest number of flightless birds (Tomlinson et al. 2024) and contains high fungal diversity. The Global Spore Sampling Projects utilises standardised air sampling methods across many countries (Ovaskainen et al. 2024), however, its focus is strictly upon atmospheric spores, and thus there lies an opportunity to expand upon this research by incorporating organismal investigations into fungal spore research and their interactions alongside air sampling. Focusing on birds specifically, there are still many other comparisons that can be made such as between feeding guilds, which may lead to novel discoveries between fungi and birds. As shown in this study, multiple organisms across taxa have relationships with fungal spores and so further studies are required to explore the connections/networks between various taxa at multiple scales and the ecological processes that can bring them together; for example, food webs and feeding habits have been identified as being important for spore dispersal (Caiafa et al. 2021; Johansson et al. 2025).

Where possible, characteristics of fungal fruiting bodies should be attributed to their presence on birds, for example, various aspects of fungal morphology may be adapted to attract birds as was showcased by Caiafa et al. (2021), where they found that fungi appeared to mimic the colour, shape and size of edible berries located within the area.

In terms of fungal‐insect interactions, there is a clear gap in the literature relating to possible insect dispersal at higher heights within the forest layers. Bracket fungi and crust fungi can be found high above the ground, but the insect assemblages that are found at these higher heights within trees require more attention. Logistically, it can be difficult to observe and/or collect samples from canopy areas, but many insects may be capable of transporting spores of crust fungi or lichenised fungi, which can be bountiful on tree branches.

Future work should also seek to standardise feather sample sizes, minimise pooling, and implement additional contamination controls (such as exposure of negative controls at the point of collection) to ensure higher accuracy and finer resolution in community profiling.

Lastly, it would be beneficial to test for spore viability when retrieving spores from vectors as this would provide evidence that spores can survive on/within vectors and be successfully transmitted from one substrate to another, this can be achieved through culturing methods, however, it should be noted that many fungal species are currently incapable of being cultured through common methods such as nutrient agar and therefore other possible substrates will need to be considered and explored. Additionally, as mycorrhizal fungi can be difficult to culture and often require specific hosts, inoculation experiments of suitable seedlings should be carried out to test for viability.

## 5 Conclusion

Overall, insects and birds appear to carry a diverse range of fungal flora, including genera that contain pathogenic species; however, insects are more likely to be beneficial vectors for ground‐based fruiting bodies due to their ability to accumulate large concentrations of fungal spores through feeding habits, whereas birds may be more adept at intercepting airborne fungi and fungal spores from a range of substrates through tactile interactions.

As species loss across a range of taxa accelerates due to habitat loss, forest fragmentation, and climate change, ecosystem stability is likely decreasing. It is therefore crucial to identify and understand the links between organisms that help to maintain biodiversity. If host‐specific interactions between fungi and insects are reduced due to insect declines, fungi may increasingly rely on other taxa as vectors. In this context, birds could play a more significant role as spore vectors, especially in fragmented and unstable forests, by dispersing fungal spores over greater distances and potentially reducing the risk of fungal genetic bottlenecks.

The interactions between fungal spores and birds or insects may strongly influence fungal community composition, particularly for mycorrhizal fungi, which are essential for tree recruitment and soil health. Our findings contribute to a growing body of evidence that animal vectors can influence fungal community assembly, with potential implications for forest ecosystem processes. While more work is needed to determine the generality of these patterns across different biomes, such studies may ultimately inform approaches to conservation and biodiversity management.

## Author Contributions

**Rohit Bangay:** conceptualization (lead), data curation (lead), formal analysis (lead), investigation (lead), methodology (lead), resources (equal), validation (lead), writing – original draft (lead), writing – review and editing (lead). **Shunsuke Matsuoka:** methodology (supporting), resources (equal), software (equal). **Nobuko Tuno:** supervision (lead), writing – review and editing (supporting).

## Funding

This work was supported by JST, the establishment of university fellowships towards the creation of science technology innovation, Grant Number JPMJFS2116 and JST SPRING, Grant Number JPMJSP2135.

## Conflicts of Interest

The authors declare no conflicts of interest.

## Supporting information


**Appendix S1:** ece372929‐sup‐0001‐AppendixS1.docx.

**Appendix S2:** ece372929‐sup‐0002‐AppendixS2.xlsx.

## Data Availability

All the required data are uploaded as Appendix S1 and supporting links. The fungal data collected from bird feathers and air samples have been deposited to BioProject accession number PRJDB35673 in the DDBJ BioProject database. Individual sequence data are available under accession numbers DRR704693–DRR704709 (Bird samples) and DRR795115–DRR795243 (Air samples), https://ddbj.nig.ac.jp/search/entry/bioproject/PRJDB35673.
