# Supplementary material for: Comparative Analysis of Fungal Spore Flora Among Birds, Insects and Air in a Temperate Japanese Forest
Source: Ecol Evol. 2026 Jan 11;16(1):e72929. doi: 10.1002/ece3.72929 (PMC12790855; doi:10.1002/ece3.72929)
Supplement: Supplementary file 1 — Appendix S1: ece372929‐sup‐0001‐AppendixS1.docx. [file ECE3-16-e72929-s002.docx]

**Appendix S1**

Table S1) Spore carrying rates of Insect collected ground-based fungal specimens. Includes 2 unidentified invertebrates.

| **Fungal Specimen** | **Examined** | **With Spores** | **% With Spores** |
| --- | --- | --- | --- |
| *Amanita eijii* | 8 | 0 | 0 |
| *Amanita spissacea* | 31 | 5 | 16.1 |
| *Amanita virgineoides* | 6 | 4 | 66.7 |
| *Amanita virosa* | 11 | 4 | 36.4 |
| *Clitocybe nebularis* | 13 | 5 | 38.5 |
| *Flammulina velutipes* | 16 | 14 | 87.5 |
| *Inonotus mikadoi* | 4 | 2 | 50 |
| *Laetiporus sulphureus* | 3 | 0 | 0 |
| *Mycena galericulata* | 7 | 6 | 85.7 |
| *Panaeolus papilionaceus* | 1 | 1 | 100 |
| *Pleurotus ostreatus* | 6 | 1 | 16.7 |
| *Russula sp* | 33 | 20 | 60.6 |
| *Russula virescens* | 2 | 2 | 100 |
| *Thelephora aurantiotincta* | 7 | 2 | 28.6 |
| *Tyromyces chioneus* | 10 | 6 | 60 |
| *Total* | 158 | 72 | 45.57 |


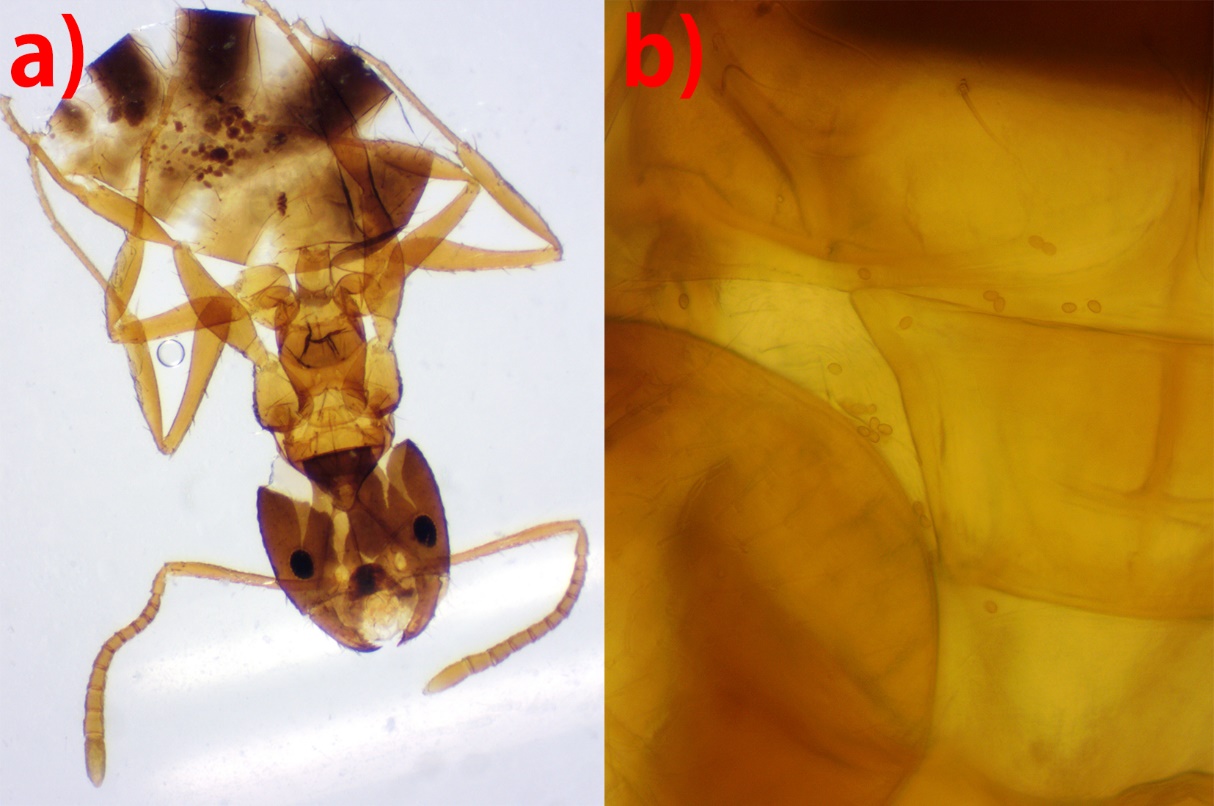


Fig. S1a) A Formicidae specimen at 100x magnification, S1b) *Inonotus mikadoi* spores within crevices between joints.


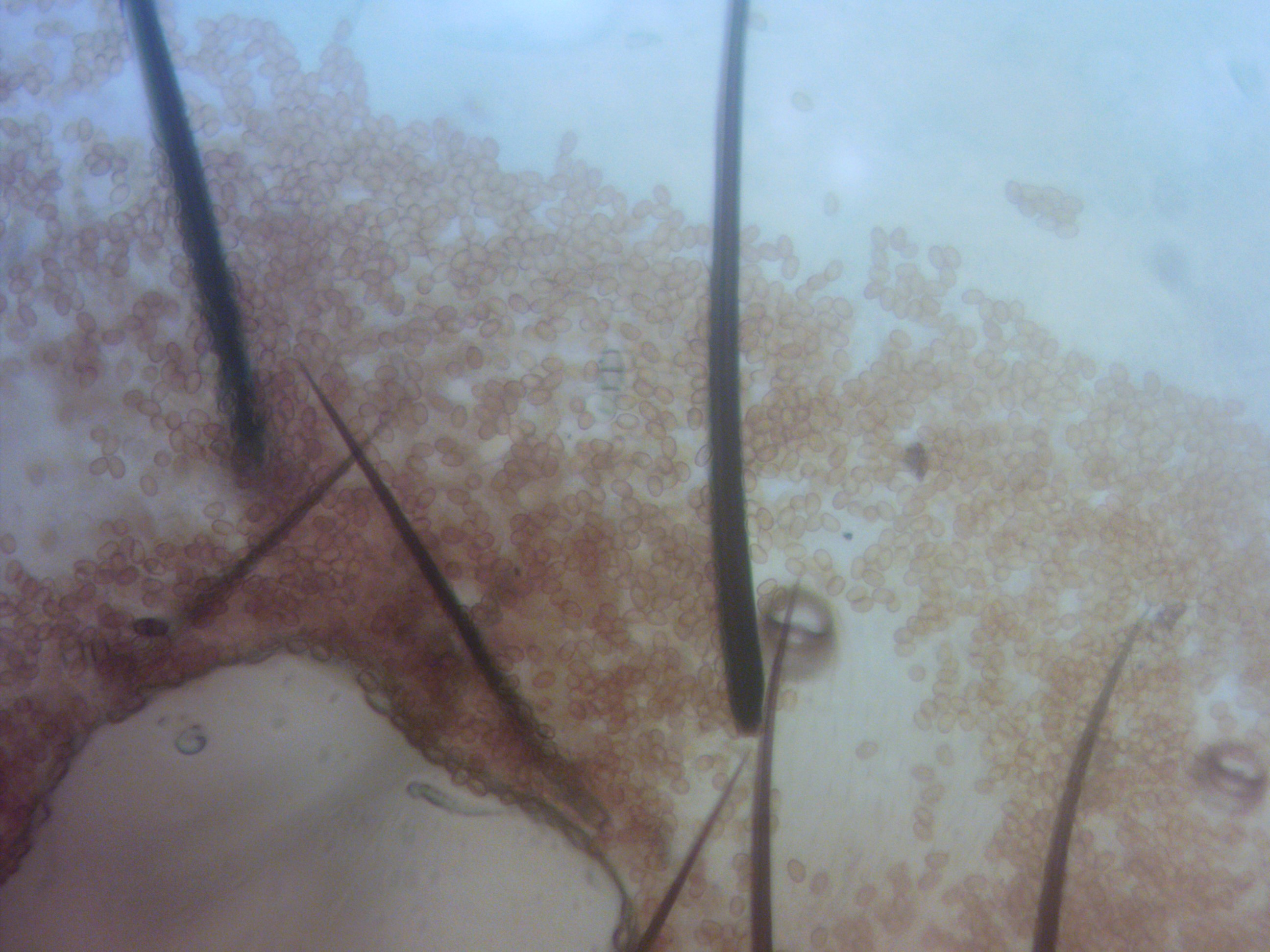


Fig. S2) Spores identified within the digestive tract of a *D. bizonata* specimen, collected from an *I. mikadoi* fruiting body.


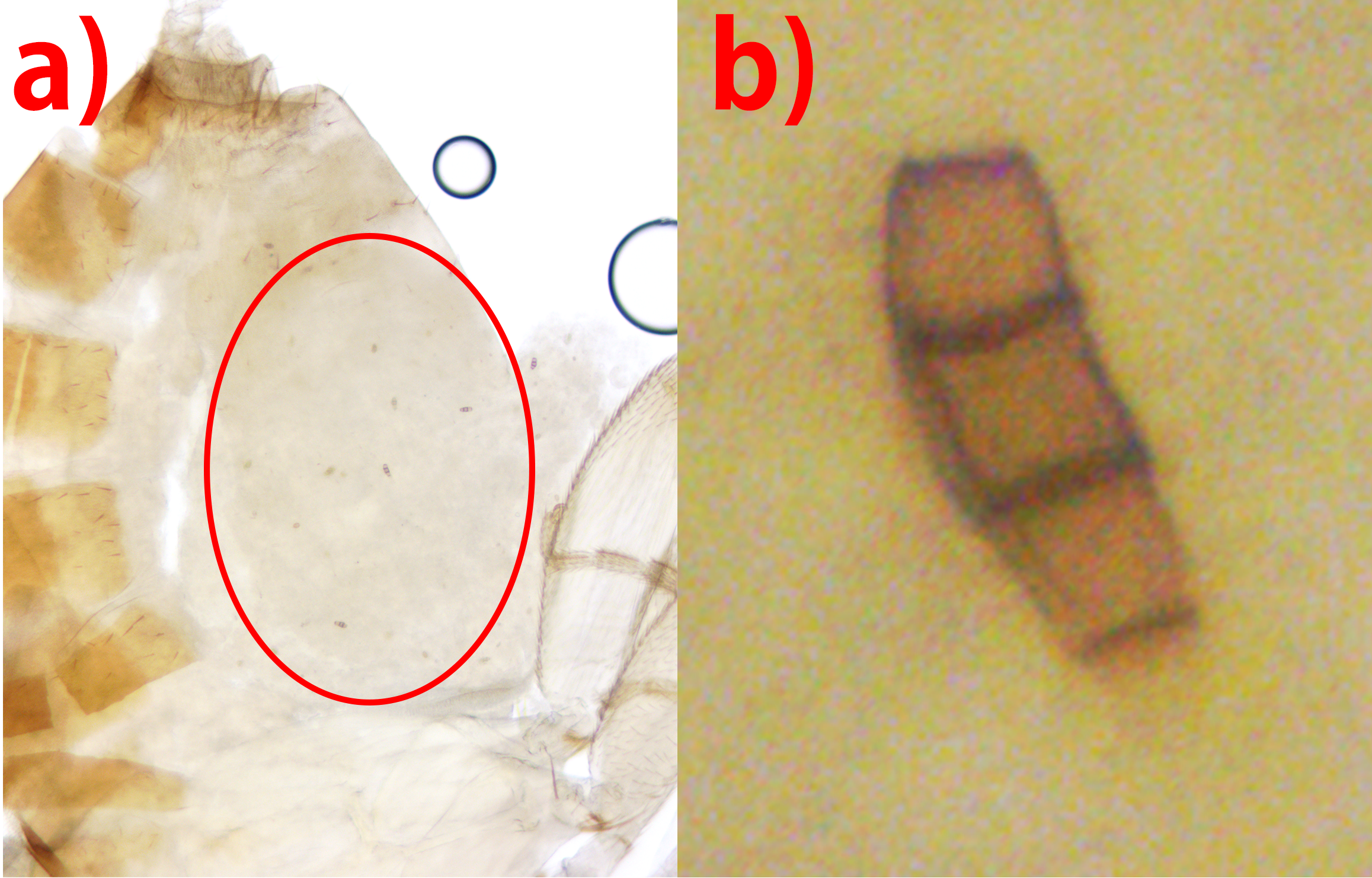


Fig. S3a) Fungal spores within the digestive tract of a Phorid. S3b) *Pestalotiopsis* spore.

Table S2) List of bird species that were captured during mist netting and whether their feathers contained spores.

| Species | English Name | Japanese Name | Collection Date | Spores |
| --- | --- | --- | --- | --- |
| *Zosterops japonicus* | Japanese White Eye | メジロ | 26/06/2024 | Yes |
| *Emberiza cioides* | Meadow Bunting | ホオジロ | 31/07/2024 | Yes |
| *Acrocephalus orientalis* | Oriental Reed Warbler | オオヨシキリ | 08/08/2024 | Yes |
| *Horornis diphone* | Japanese Bush Warbler | ウグイス | 05/11/2024 | Yes |
| *Emberiza spodocephala* | Black-faced Bunting | アオジ | 05/11/2024 | Yes |
| *Turdus chrysolaus* | Brown-Headed Thrush | アカハラ | 05/11/2024 | Yes |
| *Turdus eunomus* | Dusky Thrush | ツグミ | 05/11/2024 | Yes |
| *Fringilla montifringilla* | Brambling | アトリ | 05/11/2024 | Yes |
| *Phoenicurus auroreus* | Duarian Redstart | ジョウビタキ | 05/11/2024 | Yes |
| *Leiothrix lutea* | Red-billed leiothrix | ソウシチョウ | 05/11/2024 | Yes |
| *Emberiza rustica* | Rustic Bunting | カシラダカ | 05/11/2024 | Yes |
| *Phylloscopus xanthodryas* | Japanese leaf warbler | メボソムシクイ | 05/11/2024 | Yes |
| *Turdus pallidus* | Pale Thrush | シロハラ | 05/11/2024 | Yes |
| *Turdus obscurus* | Eyebrowed Thrush | マミチャジナイ | 05/11/2024 | Yes |

Spores were present on all examined feathers. The most abundant fungal genera found on feathers when analysed microscopically were *Penicillium* and *Cladosporium* (Fig. 4 & 5).


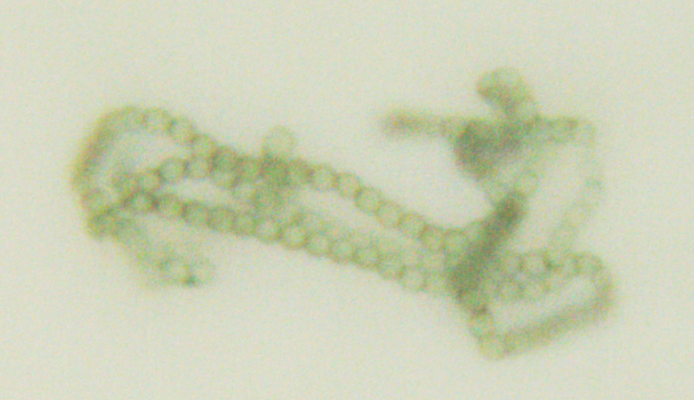


Fig. S4) *Penicillium* spores collected from the feathers of a Brown-Headed Thrush *(T. chrysolaus),* viewed at 400x magnification.


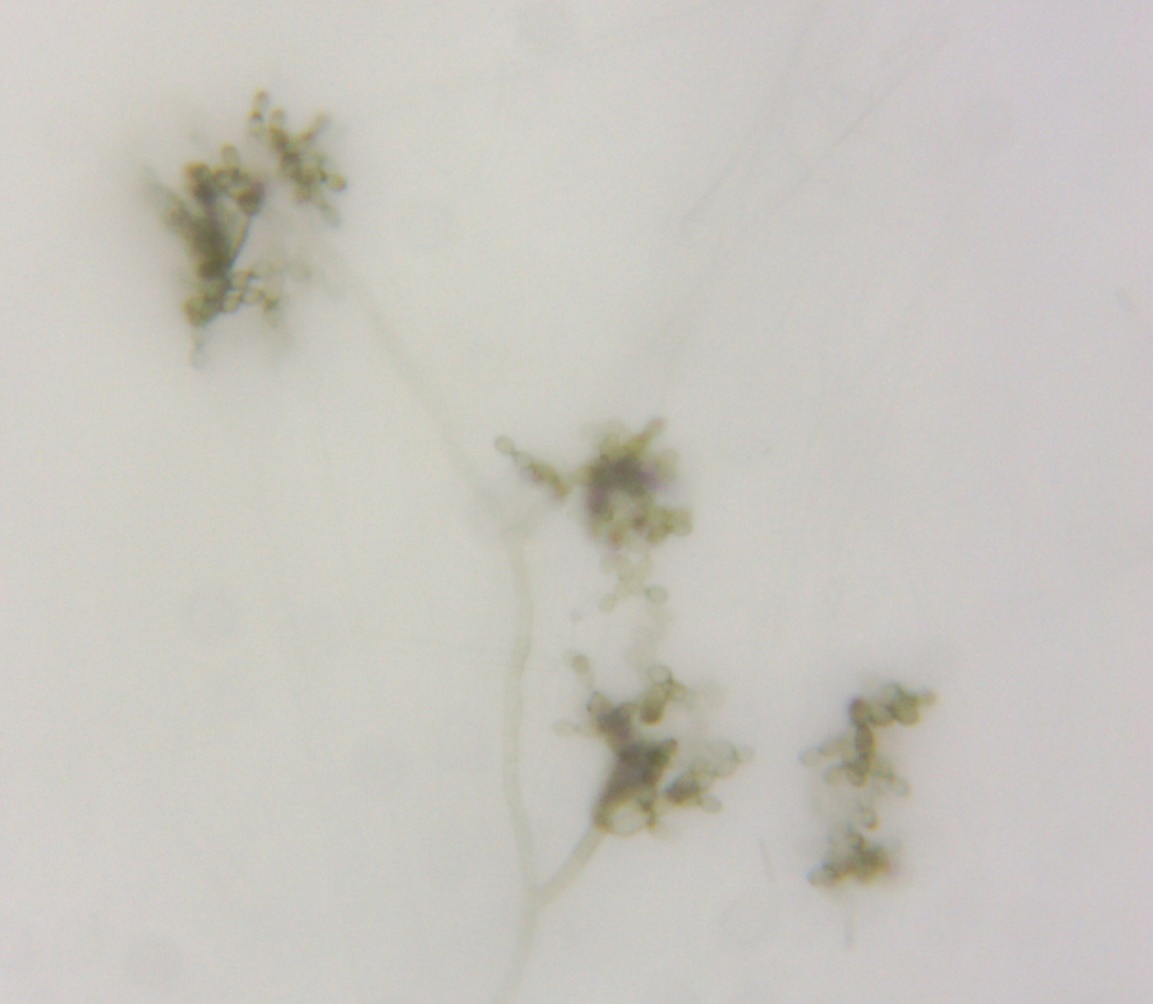


Fig. S5) *Cladosporium* spores collected from the feathers of a Japanese White Eye *(Z. japonicus),* viewed at 400x magnification.





Fig. S6) Phylum-level composition of bird-associated fungal spore flora by season, area and foraging behaviour.


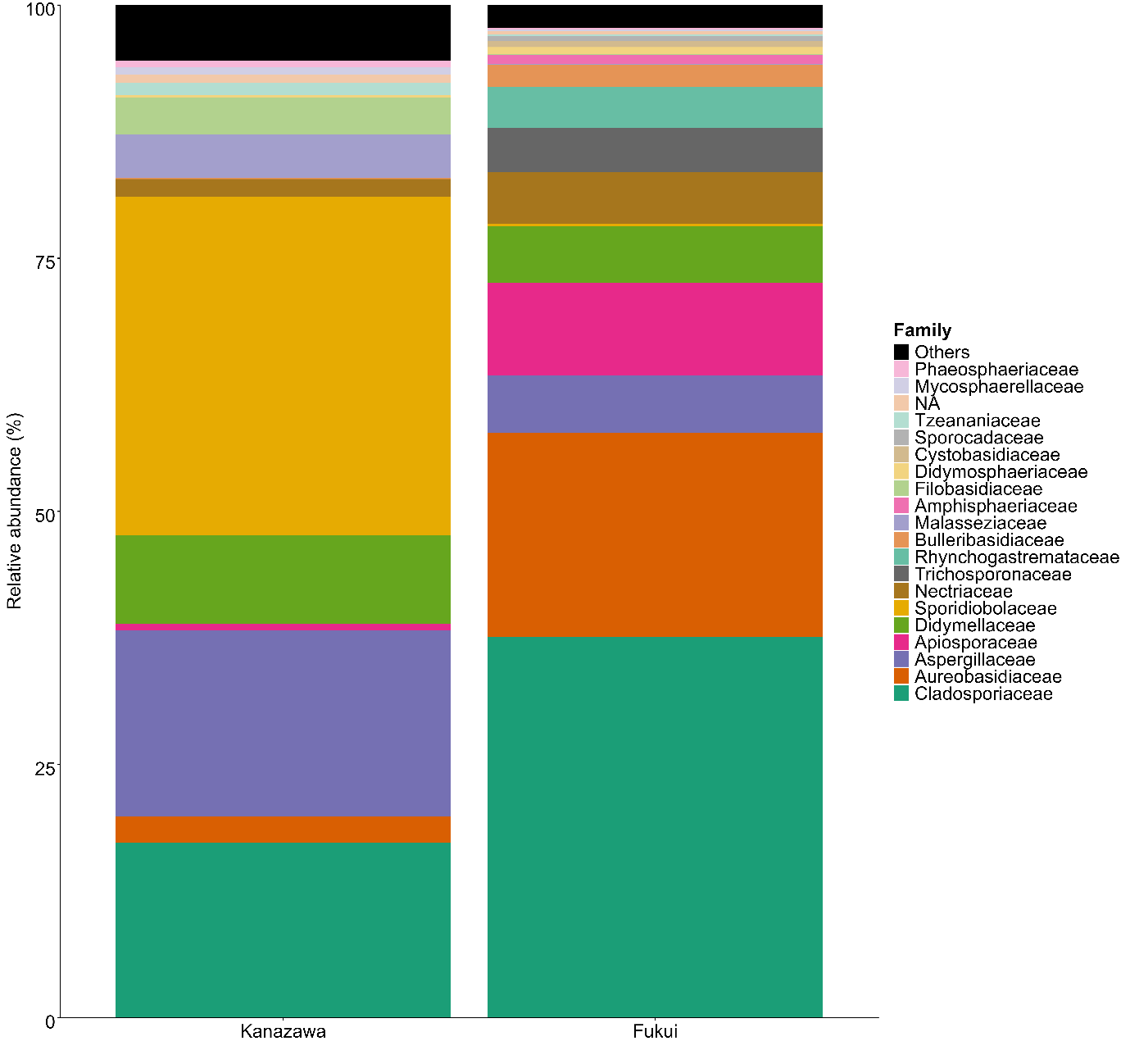


Fig. S7) Mean relative abundance across all Kanazawa and Fukui feather samples. The relative abundances between different fungal families differs between area/season.


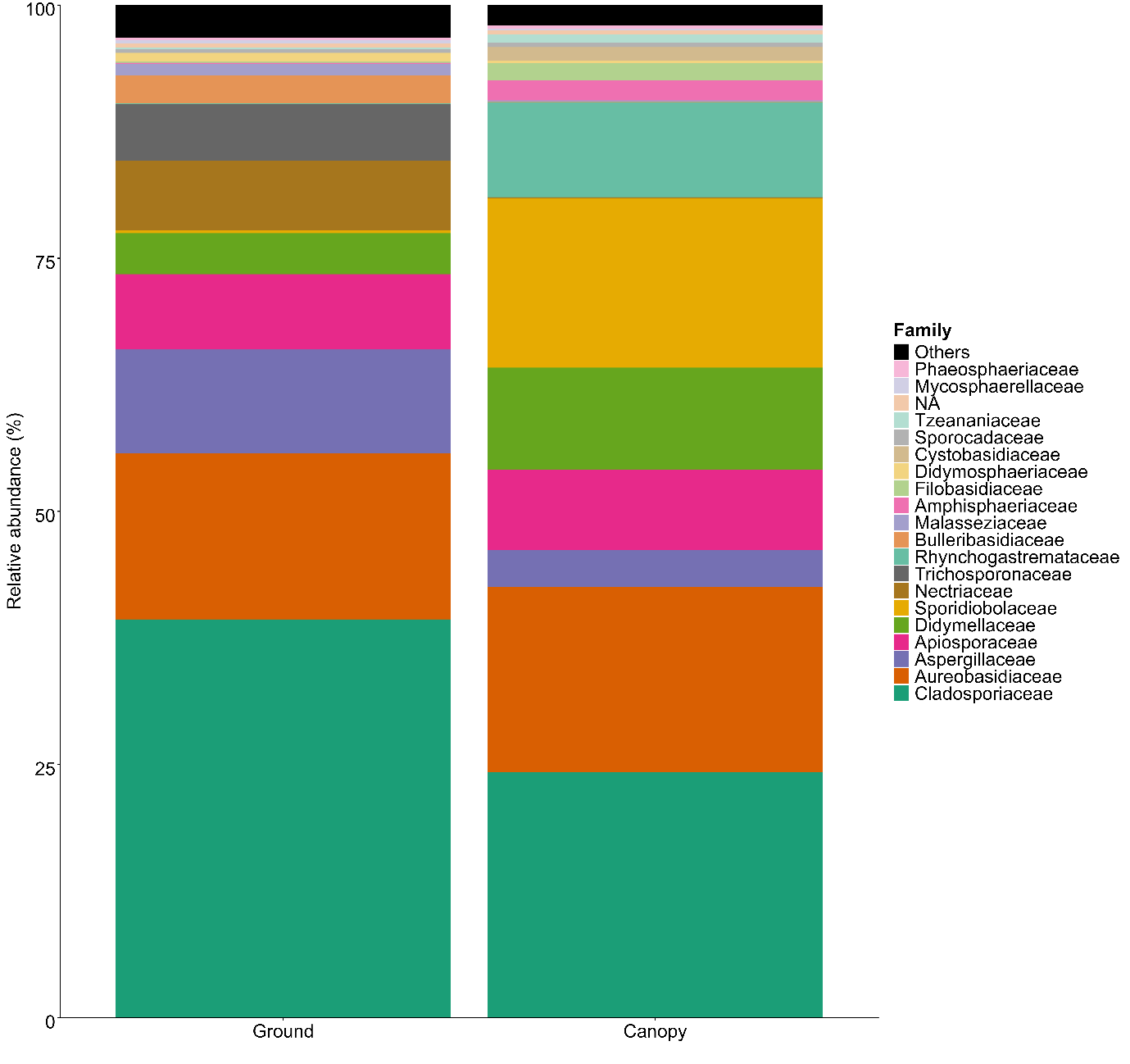


Fig. S8) Mean relative abundance of fungal families based on the foraging behaviours of the bird species.


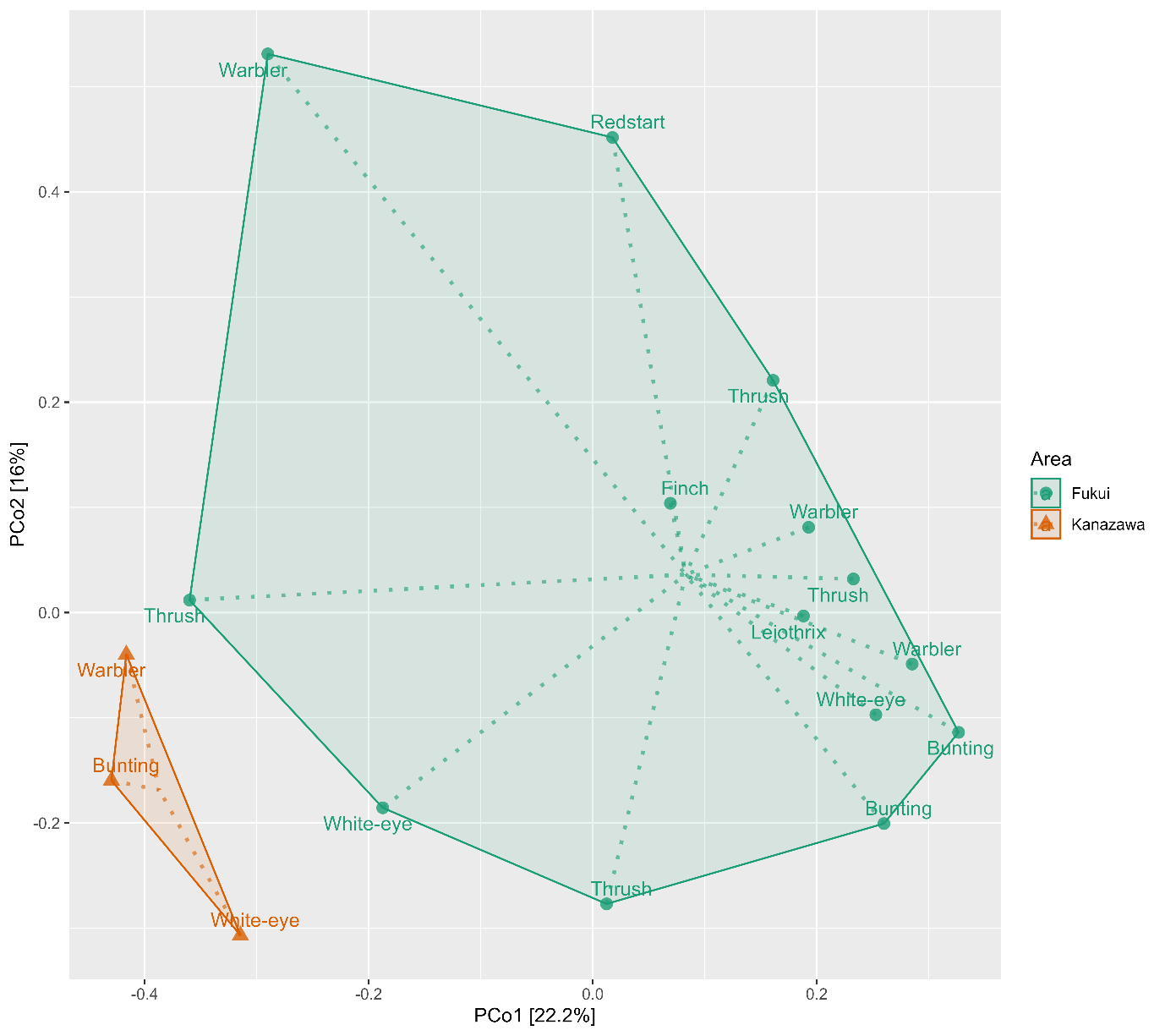
Fig. S9) Principal Coordinates Analysis (PCoA) was performed to visualise differences in fungal community composition among bird samples from Kanazawa (summer) and Fukui (autumn). The first two principal coordinate axes explained a combined total of 38.2% of the entire variance in fungal spore flora when not considering any variables.


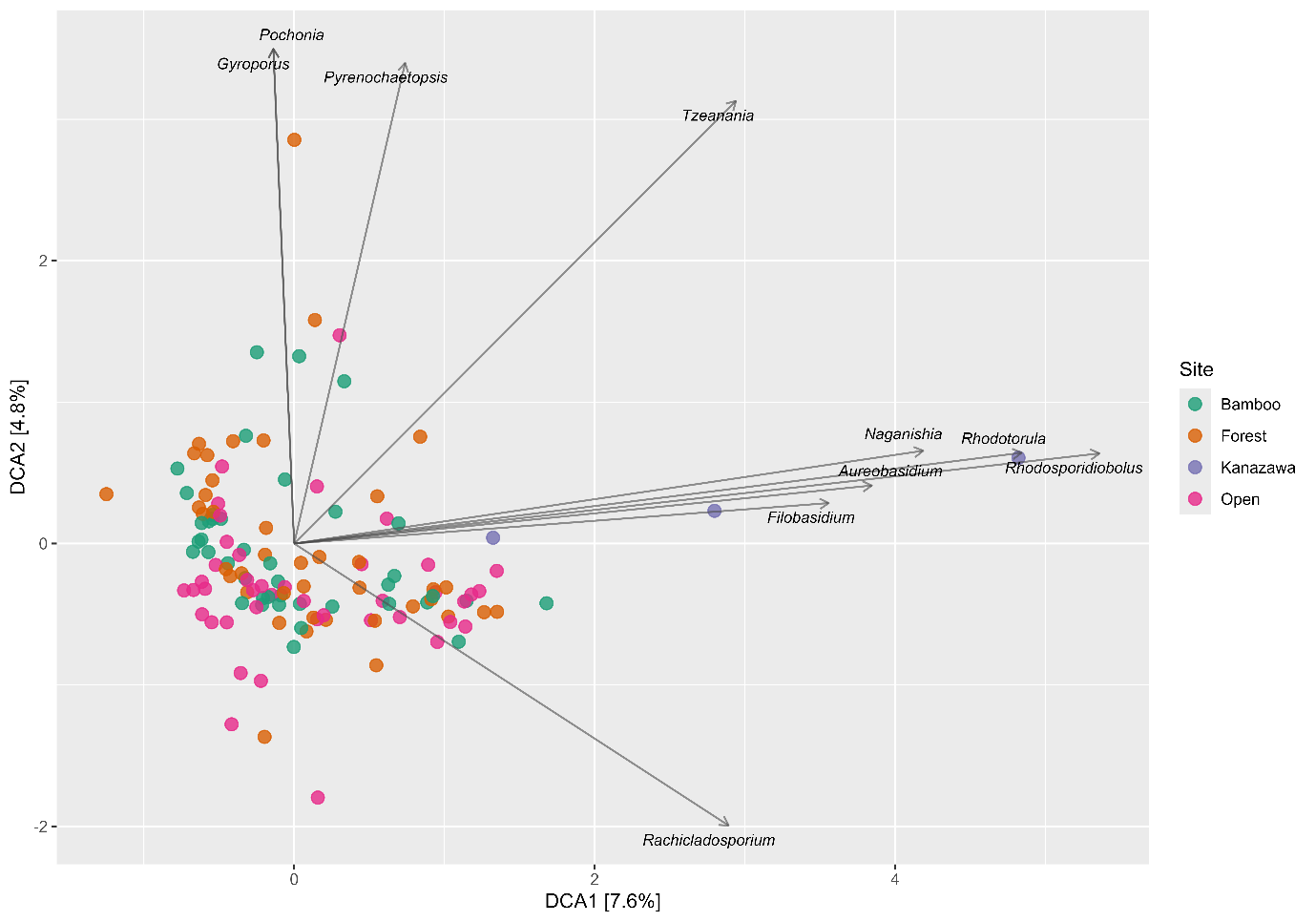


Fig. S10) DCA1 and DCA2 axes represent the main gradients of species turnover among samples. DCA1 explains 7.6% and DCA2 4.8% of the variation. This DCA shows that differences in fungal communities exist, with Kanazawa feather samples being most distinct, and highlights which fungal genera are most associated with those differences, in this case *Rachicladosporium, Filobasidium, Naganishia, Aureobasidium* and *Rhodotorula*, are all strongly associated with feather samples. These results are based on RDP taxonomic assignment.


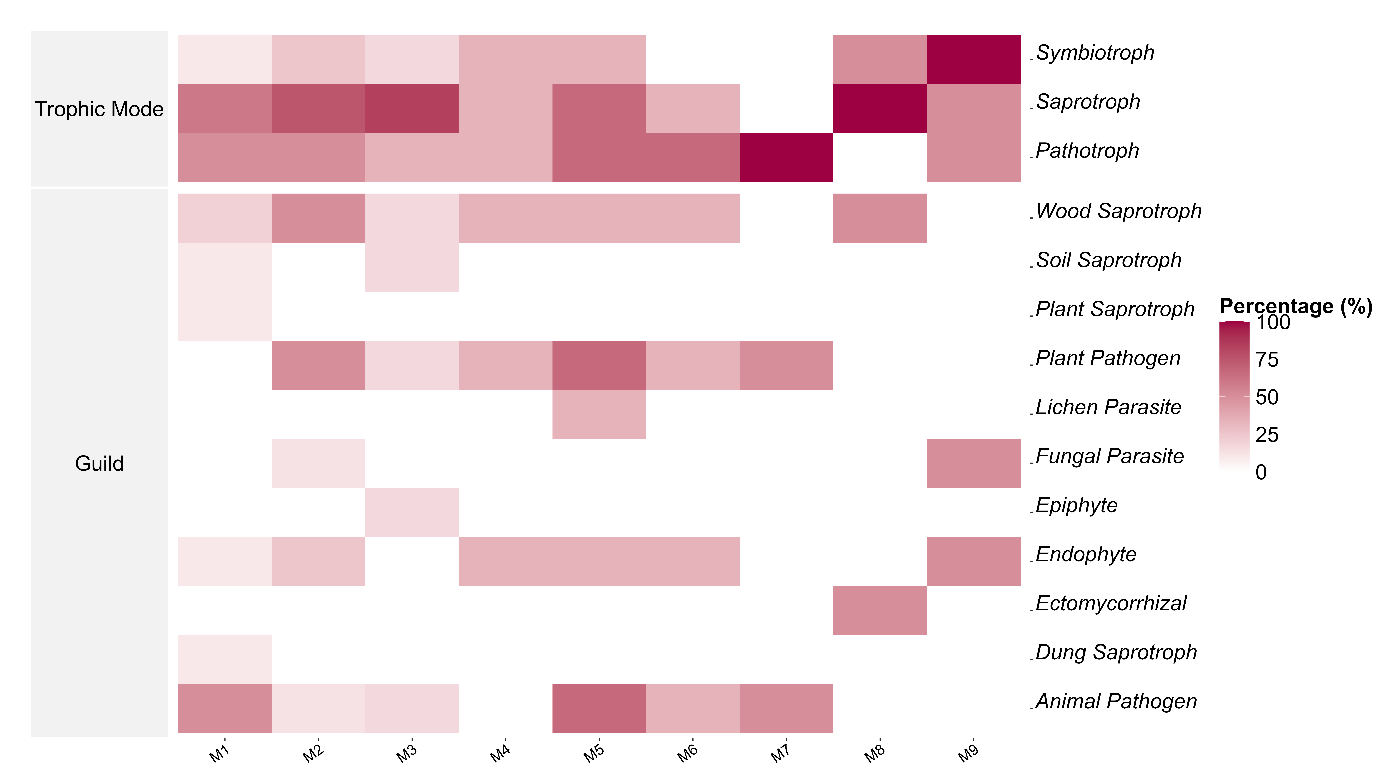
Fig. S11) Heatmap of fungal trophic modes and ecological guilds in bird-associated fungal spore flora (FUNGuild). Trait analysis based on Claident taxonomic species assignment (39 species), associated the species to symbotrophic, saprotrophic or pathogenic ecological guilds.
